# Optimal dose and type of exercise to improve depressive symptoms in older adults: a systematic review and network meta-analysis

**DOI:** 10.1186/s12877-024-05118-7

**Published:** 2024-06-07

**Authors:** Lili Tang, Lin Zhang, Yanbo Liu, Yan Li, Lijuan Yang, Mingxuan Zou, Huiran Yang, Lingyu Zhu, Ruihong Du, Ye Shen, Haoyu Li, Yong Yang, Zhijun Li

**Affiliations:** 1 Department of Epidemiology, School of Public Health, Beihua University, No. 3999 Binjiang Road, Jilin, 132000 Jilin Province China; 2 Department of Rehabilitation, West China Hospital Sichuan University Jintang Hospital, Chengdu City, Sichuan Province China; 3 The Pathophysiology Department of Basic Medical College, Beihua University, Jilin City, 132000 Jilin Province China; 4 Basic Medical School Biochemistry, Beihua University, 132000, Jilin City, Jilin Province China; 5 National Energy, Jilin, Jilin Province China; 6 Shenyang Railway Disease Control Center, Jilin, Jilin Province China; 7 Institute for Brain Sciences Research, School of Life Sciences, Henan University, Kaifeng, 475001 Henan Province China

**Keywords:** Exercise, Dose–response relationship, Older adults, Depressive symptoms

## Abstract

**Background:**

Depression is a prevalent issue among older adults, affecting their quality of life and overall well-being. Exercise is an effective means of relieving depressive symptoms in older adults, but the optimal dose for different exercise types remains unclear. As such, the aim of this meta-analysis was to examine the dose–response relationship between overall and specific types of exercise with depression symptoms in older adults.

**Methods:**

This systematic review and network meta-analysis included a search of PubMed, Medline, Embase, PsycINFO, Cochrane library, and Web of Science for randomized controlled trials of exercise in older adults with depression symptoms from inception to 15 July 2023. Comprehensive data extraction covered dose, treatment regimen, demographics and study duration. Dosage metrics, encompassing METs-min/week, were scrutinized in correlation with the Minimal Clinically Importance Difference (MCID).

**Results:**

A total of 47 studies involving 2895 participants and 7 kinds of exercise were included in the review. Without considering the dose, the results of our network meta-analysis indicated that Walking was the most effective in alleviating depression in older adults, in addition to Aerobic exercise (AE), Yoga, Qigong, Resistance training (RT), and Tai Chi (TC), which were equally effective. However, the results of the dose–response analysis found that Aerobic exercise was most effective at a dose of 1000 METs-min/week. It is noteworthy that Walking is significantly effective in alleviating depressive symptoms in older adults at very low doses. In terms of clinical benefits, we found that overall exercise doses in the range of 600 ~ 970 METs-min/week were clinically effective. Considering the specific types of exercise, Aerobic exercise, Resistance training, Walking, and Yoga were found to be effective at doses ranging from 820 ~ 1000 METs-min/week, 520 ~ 1000 METs-min/week, 650 ~ 1000 METs-min/week, 680 ~ 1000 METs-min/week, respectively. At the same time, we found that when the age exceeded 81 years, even when participating in exercise, it did not achieve the effect of alleviating depressive symptoms in older adults.

**Conclusions:**

In conclusion, including Walking, AE, Yoga, Qigong, RT, and TC, effectively alleviate depressive symptoms in older adults. Furthermore, we established statistically and clinically significant threshold doses for various exercise types. Early initiation of exercise is beneficial, but its efficacy diminishes from the age of 80, and beyond 81, exercise no longer significantly alleviates depressive symptoms.

**Supplementary Information:**

The online version contains supplementary material available at 10.1186/s12877-024-05118-7.

## Background

Depression is a prevalent issue among individuals aged 60 years and older. It is closely linked to a decline in their overall quality of life, leading to increased physical limitations, heightened reliance on healthcare services, notably frequent hospital readmissions, and even a rise in mortality rates [1, 2]. Depression contributes significantly to the overall burden of disease in China, accounting for more than 6.9% to 7.8% [3]. In the United States alone, the economic impact of depression is substantial, estimated to exceed $210.5 billion annually [4]. While antidepressant medication is frequently the initial treatment choice for depression in older adults [5], it is important to acknowledge the associated risks in this age group [6, 7]. Alarmingly, nearly half of older adults treated with antidepressants do not achieve full symptom remission [8, 9].

Identifying potentially accessible and cost-effective health and lifestyle behaviors that can help mitigate risk factors for depressive symptoms and disorders is of paramount importance [10]. A meta-analysis summarizing 111 prospective cohort studies involving 3 million adults revealed that habitual and progressively increasing levels of moderate to vigorous physical activity, regardless of the global region, gender, age, or duration of follow-up, exhibited a negative correlation (OR: 0.69, 95% Crl (0.63 to 0.75)) with the incidence of adult depression and the onset of subclinical depressive symptoms in observational studies [11]. In a recent cohort study involving 4,016 elderly individuals, at each time point over a 10-year period, a negative dose–response relationship was observed between moderate to high-intensity physical activity and the presence of depressive symptoms and severe depression. It was noted that a minimum weekly exercise dosage of 400 MET*min was required for effectively reducing the occurrence of depressive symptoms. [10]. Hence, adopting physical activity or exercise as a regular habit proves to be a cost-effective and efficient healthy lifestyle choice for preventing depression in the older adult.

Regarding the efficacy of exercise in alleviating depressive symptoms in the older adults, results from meta-analysis as far back as a decade ago indicated that exercise is an effective means of mitigating depression [12, 13]. Recent evidence-based medicine has been dedicated to exploring which type of exercise is more effective in alleviating depressive symptoms in the older adults. Miller, et al. (2020) [14] combined the results of 15 randomized controlled trials (RCTs) involving 596 participants, revealing that mind–body exercises are the most effective in relieving depressive symptoms (large effect size: -0.87 to -1.38), followed by aerobic exercises and resistance training. However, another commonly effective mind–body exercise, yoga [15–18], was not included in the comparative efficacy assessment. At the same time, it remains uncertain whether combining the effect sizes of low-intensity exercises such as walking with higher-intensity aerobic exercises (e.g., dancing or running) accurately reflects the true efficacy of aerobic exercise in alleviating depressive symptoms in older adults.

Laird, et al. (2023) [10] evaluated the effects of different doses of physical activity on the risk of depression in 4,016 older people, and the results showed that as the dose of physical activity increased, the adjusted incidence rate ratio (AIRR) of depression decreased (compared to no exercise, 1–600 METs*min/week (AIRR: 0.96, 95% CrI (0.91 to 1.01); 600–1200 METs*min/week (AIRR: 0.92, 95% CrI (0.86 to 0.98); 1200–2400 METs*min/week (AIRR: 0.82, 95% CrI (0.77 to 0.87); ≥ 2400 METs*min/week (AIRR: 0.77, 95% CrI (0.73 to 0.80))). However, the optimal dose of specific exercise types to alleviate depressive symptoms in older adults remains unknown. Moreover, when conveying research findings to various stakeholders such as patients, caregivers, and clinicians, it becomes imperative for researchers to elucidate both the statistical and clinical significance of their results. This task can be particularly challenging when clinical scales are employed to measure changes in health status. The reason is that, even when statistical significance is established for a specific treatment approach, its clinical significance may remain uncertain [19, 20]. The clinical significance of a change measured on a scale hinges on understanding the minimal clinically important difference (MCID) associated with that scale. Clinicians, patients, and researchers concur that an observed difference in outcomes should surpass this threshold [21]. In situations where the MCID for a particular scale remains unidentified, clinicians may face challenges in discussing the clinical significance of study outcomes with patients. The absence of this pivotal information can render the shared decision-making process between clinicians and patients less effective. This insufficiency in shared decision-making can adversely impact patients, as it may hinder their understanding of the potential benefits and risks associated with different treatment options.

Utilizing an innovative meta-analysis approach, specifically the model-based dose–response network meta-analysis within the Bayesian framework, and drawing upon data from existing RCTs, this study ranks the effectiveness of seven exercise types (Aerobic Exercise (AE), Mixed Physical Activity (Mix), Qigong, Resistance Training (RT), Tai Chi (TC), Walking, and Yoga) in relieving the depressive symptoms in older adults. Furthermore, it delves into assessing the dose–response relationship between various exercise types and depressive symptoms. In an effort to enhance the clinical interpretation of our study, we also estimated the required dose range for each exercise types to attain a MCID in depressive symptoms.

## Methods

This pre-registered systematic review with network meta-analysis (PROSPERO reference number # CRD42023457294) was reported following the PRISMA checklist [22].

### Search strategy

We conducted a systematic search in PubMed, MEDLINE, Embase, PsycINFO, Cochrane Central Register of Controlled Trials (CENTRAL), and Web of Science from their inception date to July 15, 2023, with no language restrictions. The specific search strategies, including search terms, dates, and process, are shown in Supplementary File 1. The reference lists of relevant articles and reviews were also screened for additional studies. Title/abstract and full-text screening were conducted independently and in duplicate by investigators, with disagreements resolved by discussion or adjudication by a third author.

### Study selection

The inclusion criteria were based on the PICOS (participants, interventions, comparators, outcomes, and study design) approach [23]. (1) the participants were diagnosed with clinical depression at baseline according to a structured diagnostic interview, and the mean age ≥ 60 years; (2) the exercises were divided into 7 types according to their content; (3) the control group (CON) included non-intervention, regular daily activities, wait-list, health education or usual care. Besides, for head-to-head studies, the comparator may be any of the 7 exercise types; (4) studies should include the outcome measures of interest: depressive symptoms; (5) in the study design, we included published RCTs (individual design, cluster design, or the first half of crossover). We excluded studies if (1) the acute effects of a single session on depressive patients; (2) studies that did not clearly describe the types of exercise and the dose of treatment; (3) participants were not assessed clinically depressed at baseline; (4) depressive symptoms were not assessed as an outcome, based on change scores between baseline and follow-up; (5) the sample had a mean age below 60 years.

### Data extraction

After all relevant articles were searched in the mentioned databases; they were stored in an EndNote X9 reference manager. Two authors independently extracted data from studies that met the inclusion criteria and disagreements were resolved by consensus between all authors. Relevant publication information (i.e., author, title, year, journal), number of patients, patient characteristics (e.g., age and sex), interventions considered and outcome measures (clinical cut-off scores) were extracted. In the process of extracting data, if the original study reported a standard error in the experimental and control groups, the standard deviation was calculated by the formula: standard deviation (SD) = standard error (SE) × √n. In cases where both standard deviation (SD) and other direct measures are unavailable, we will derive the SD using methods based on confidence intervals, t-values, quartiles, ranges, or p-values. These methods are outlined in detail in Sect. 7.7.3 of the Cochrane Handbook for Systematic Reviews of Interventions. If the necessary data cannot be obtained by the above methods, we will write to the authors to inquire about the raw data. If there is no reply, we will exclude the article. In addition, in order to reduce the risk of type 1 error, we did not extract the mid-test data during the data extraction process, and only merged the post-test data [24].

### Risk of bias and quality of evidence

Three reviewers assessed and rated the studies according to the Physiotherapy Evidence Database (PEDro) scale, based on the list of Delphi [25].The PEDro scale includes 11 items with three items from the Jadad scale [26] and nine items from the Delphi list [25]. Interrater reliability was shown to be fair to good (Intraclass Correlation Coefficient = 0.68). A total PEDro score is achieved by adding the ratings of items 2 to 11 for a combined total score between 0 to 10, and < 4 are considered ‘poor’, 4 to 5 are considered ‘fair’, 6 to 8 are considered ‘good’, and 9 to 10 are considered ‘excellent’[27, 28]. We examined the confidence of evidence using the CINeMA (Confidence of Network Meta-Analysis) web application, which allows the confidence of the results to be graded as (high), (moderate), (low), and (very low) [29].

### Data coding and management

We categorized interventions into three levels [30]: first, interventions were coded as “Exercise” and “CON” (first level). At second level, the interventions were coded according to their main exercise type: AE, Mix, Qigong, RT, TC, Walking, Yoga, and CON. Finally, interventions were coded at the intersection of specific type and dose—defined as the energy expenditure (i.e., Metabolic Equivalent of Task, MET) that results from the product of the duration, frequency, and intensity of a certain type of exercise [31, 32]; and expressed as METs-min per week. For example, 750 METs-min per week of AE. In order to better promote the connectivity of the network, we performed an approximate value of 0 (CON), 250, 500, 750, 1000, or 1200 METs-min per week for the exercise dose, which has been adopted in previous studies [30], and which is a necessary step for the network meta-analysis [33].

### Data synthesis

#### For network meta-analysis

A network plot was drawn using Stata (release 14, Stata- Corp LLC, TX, USA) to present the geometry of the network of comparisons across trials to ensure the network meta-analyses were feasible. Based on the R statistical environment (V.4.2.2, www.r-project.org), we used the gemtc and rjags packages to perform Bayesian network meta-analyses to compare the effects of different exercises because it calculates the posterior distribution of the parameters using the data to update prior information and is more common than frequentist approaches. Markov chains were used to generate samples. Model convergence was assessed using the Brooks-Gelman-Rubin plots method. The effect sizes measure chooses the standardized mean difference (SMD) of the change score (endpoint minus baseline score) because the studies use different rating scales or units of the outcome, and 95% credible intervals (CrI) were used to assess the credibility of our estimates. A random-effects model was used to combine data, and the surface under the curved cumulative ranking probabilities (SUCRA) was used to rank the treatments. The transitivity assumption was evaluated by comparing the distribution of potential effect modifiers (the publication year, mean age, sample size, percentage female, exercise period, and exercise dose (METs-min/week)) among studies grouped by comparison (Supplementary File 2). Besides, we evaluated the robustness of the treatment effects for the outcomes in the network meta-regression through the publication year, mean age, sample size, percentage female, exercise period, and exercise dose (METs-min/week). We used the tau square (τ^2^) test and I^2^ to analyze the statistical heterogeneity between the studies. We evaluated consistency statistically using the design-by-treatment test [33] and by separating indirect from direct evidence (SIDE test) [34] using the R netmeta package. We compared the adjusted funnel plot to assess the risk of publication bias under specific circumstances [35], and Egger’s test suggestive of publication bias when *p* < 0.05.

#### For dose–response

The MBNMAdose package based on R statistical environment (V.4.2.2, www.r-project.org) was used to perform random-effects Bayesian Model-Based Network Meta-Analysis (MBNMA)[36] to summarize the dose–response association between exercises dose and depressive symptoms. First, the connectivity is a key assumption in network meta-dose analysis, and evidence of un-connectedness may lead to low statistical power and misleading results [37]. By drawing treatment-level and agent-level network plots, we verified that the connectivity in this study (Supplementary File 8, Fig. 8.1–2). We analyzed the data with the consistency model and the unrelated mean effect model, and compared the differences in the deviation, the number of estimated parameters in the network, and the Deviance Informative Criterion (DIC) indicators of the two models. If these are similar, it means that our research has good consistency [38] (Supplementary File 8, Table 8.1). We assessed transitivity via MBNMA node-splitting approach. This method splits and compares contributions for a particular treatment contrast into direct and indirect evidence [39]. Similar effects denote good transitivity (Supplementary File 8, Table 8.2, Fig. 8.3). In order to better find the relationship between exercise dosage and elderly depressed patients, a series of nonlinear and linear models were used. Next, we derived and compared different fit indices and corresponding deviance plots across all estimated models [40]. Restricted cubic splines yielded the best fit in all cases and were therefore used to assess the non-linear dose–response associations (Supplementary File 9, Table 9.1). According to the model with the best fit and biological plausibility [41], we placed three knots at the 10th, 50th and 90th percentile of the treatment dose. Departure from linearity was assessed using a Wald test [42].

#### For clinical effect

To further enhance the clinical utility of our results, we estimated the dose (or range of doses) at which interventions were able to achieve the minimum clinically important difference (MCID) [43]. We used a distribution-based approach to derive a pooled MCID for the 15-item Geriatric Depression Scale (GDS-15) [44]. In this study, the GDS was estimated to be 1.3 points at 0.4 SD, 1.6 points at 0.5 SD. We then calculated the pooled effect size (SMD) of the studies that at least achieved the estimated pooled MCID. Finally, we predicted at which dose(s) of treatment these effects were achieved for each type of intervention.

## Results

**Fig. 1:**
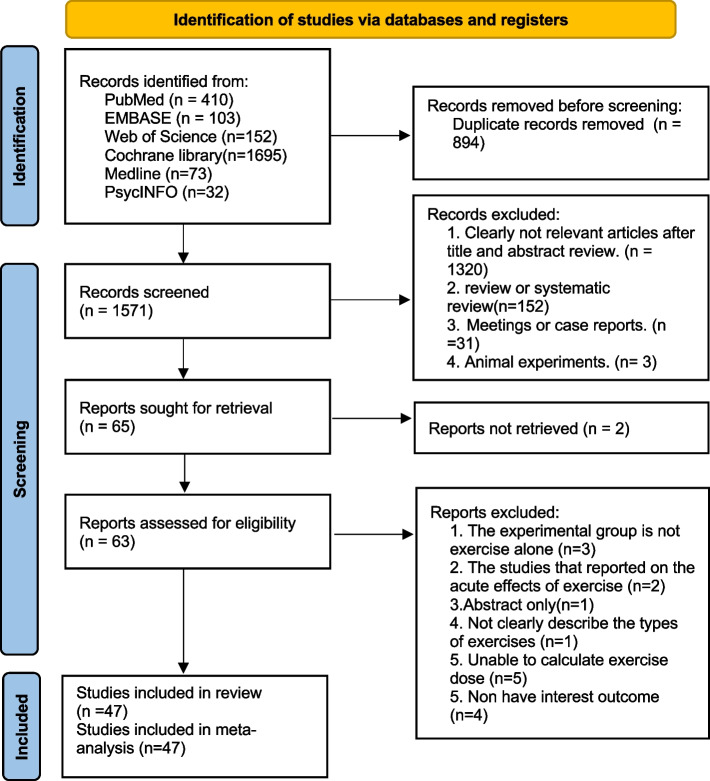
PRISMA Flow diagram of the search process for studies

Overall, 2465 records were identified through the initial electronic searches. After removing duplicates, 1571 records were screened for titles and abstracts and 63 full-text articles were screened for eligibility. In total, 47 studies involving 2895 participants (1606 treatments and 1289 controls) were included in the review (Fig. 1).

### Characteristics of included studies

The characteristics of included studies were shown in (Supplementary File 3). A total of 328 participants across 10 studies underwent AE, 11 studies with 340 participants underwent Mix, 5 studies with 179 participants underwent Qigong, 11 studies with 254 participants underwent RT, 5 studies with 81 participants underwent TC, 7 studies with 227 participants underwent Walking, and 5 studies with 173 participants underwent Yoga. The sample size of the included studies ranged from 5 to 83, with a median of 23. The mean age ranged from 59.6 to 87.9, with a median of 72.58. The year of publication ranged from 1990 and 2023, with a median of 2011. For specific exercise variables, the median reported period was 12 weeks (range 2 to 26), frequency was 3 times (range 1 to 5), and single session time was 45 min (range 20 to 70). We also reported the tools used to assess depressive symptoms in the included studies and their cut-offs for depressive symptoms. Among them, GDS-15 was the most used (15 studies). The literature quality of the 21 included studies (44.7%) was fair (PEDro score: 4–5), and 26 included studies (55.3%) was good (PEDro score: 6–8). Overall, the literature quality was low to moderate (mean PEDro score: 5.74 ± 0.99) (Supplementary File 4).

### Network meta-analysis

**Fig. 2:**
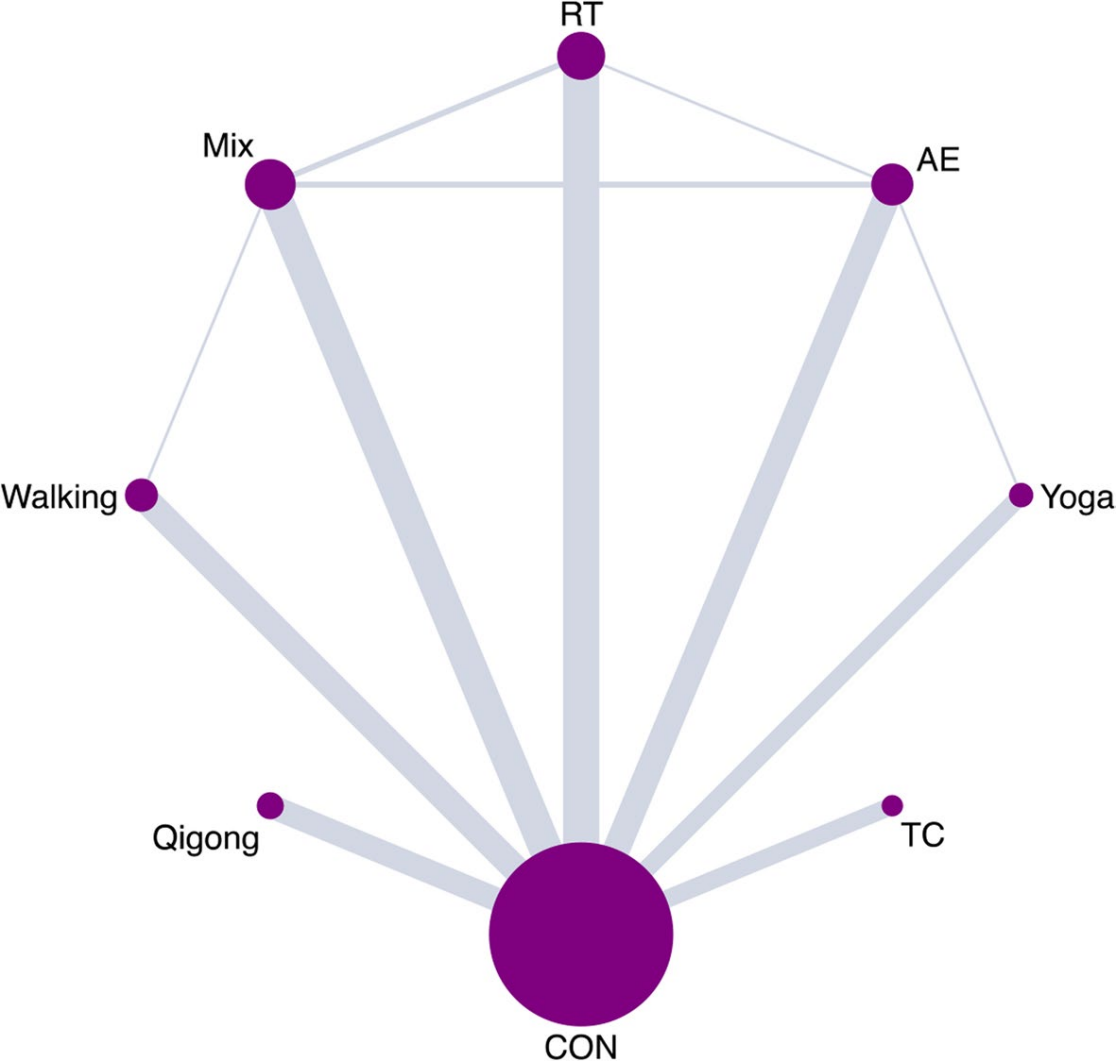
Network plot. AE Aerobic Exercise, Mix Multicomponent Exercise Program, RT Resistance Training, TC Tai Chi

**Fig. 3:**
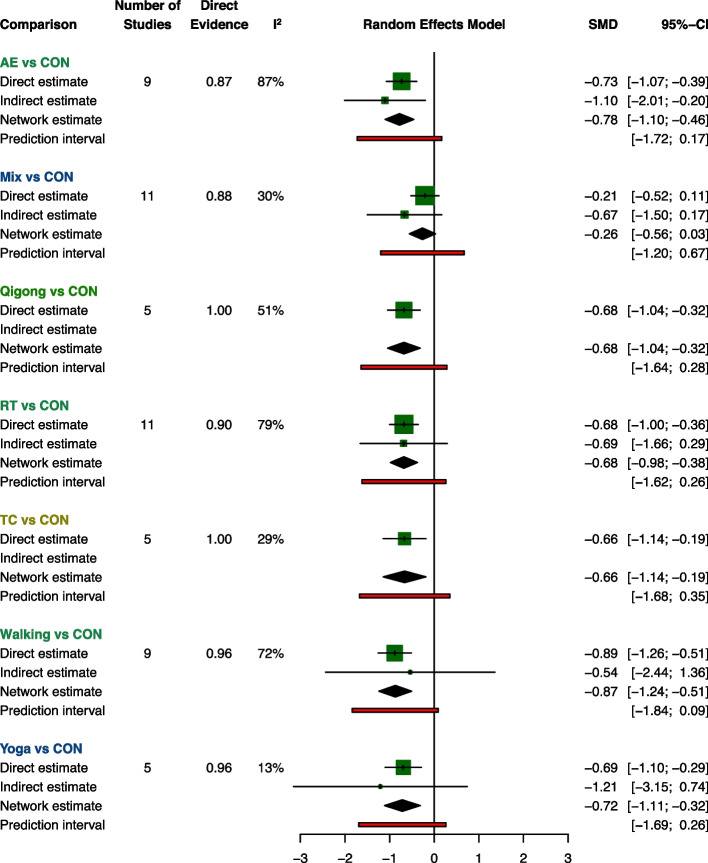
Summary network meta-analysis results for each exercise type compared with controlled group. The coloring of the exercise type represents the confidence of the evidence, green is high, blue is moderate, yellow is low, and red is very low (non-available)

Figure 2 showed the direct comparison and sample size distribution between the exercise types. Compared with the CON, 6 (85.7%) of 7 exercise types significantly relieved symptoms of depression, and the SMDs (95% CrI) ranged between -0.87 (-1.24; -0.51) for Walking to -0.66 (-1.14; -0.19) for TC (Fig. 3). The results of pairwise comparisons showed that Walking, AE, and RT were significantly better than Mix in relieving depression symptoms, and Walking ranks first (SUCRA: 0.81) (Table 1).

**Table 1:**
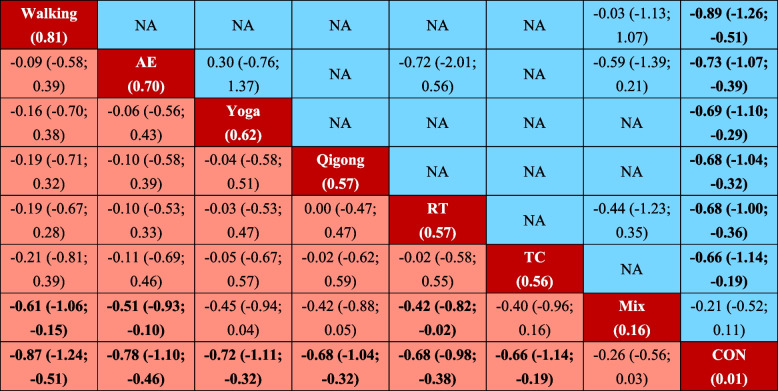
League table of depression symptoms

All results are presented in the form of SMD (95% CrI). Exercise types are ranked according to the surface under the curve cumulative for balance starting with the best from left to right. The results of the network meta-analysis are showed in the lower left part, and results from pairwise comparisons in the upper right half (if available). Cells shown in bold indicate significant results. *NA* not available, *SMD* standardized Mean Difference, *CrI* Credible Interval, *AE* Aerobic Exercise, *CON* Control group, *Mix* Mixed exercise, RT Resistance Training, *TC* Tai Chi

Heterogeneity results showed moderate to high (I^2^ = 70.8%, τ^2^ = 0.1962, *p* < 0.0001). The results of network meta-regression showed that participant's age (Beta: 0.36, 95% Crl (0.05; 0.68)) and exercise dose (Beta: -0.51, 95% Crl (-0.80; -0.22)) significantly affected exercise efficacy in reducing depressive symptoms, especially exercise dose, adjusted heterogeneity decreased by 30% (Table 2). The results of design-by-treatment interaction test showed that global inconsistency was not significant (Q = 12.84, τ^2^ = 0.2064, *p* = 02327). The SIDE test of depressive symptoms showed that the percentage of comparisons with evidence of inconsistency was 0% (Supplementary File 5). Additionally, our comparison-adjusted funnel plot had good symmetry for depressive symptoms, and the results of Egger’s test (*p* = 0.059) showed that no small study effect was found (Supplementary File 6). The confidence of the evidence of 57.1% for comparison with CON was high (AE, Qigong, RT, and Walking), 28.6% was moderate (Mix, and Yoga), and 14.3% was low (TC). For the comparisons of two exercise types, the confidence of evidence of 42.9% was high, 23.8% was moderate, 19.0% was low, and 14.3% was very low (Supplementary File 7).

**Table 2:** Network Meta-Regression. Below we present the results from the changes in heterogeneity in each meta-regression model

Covariate	Shared beta (median and 95% CrI)	Heterogeneity standard deviation (median and 95% CrI)	% of variance explained
None	-	0.49 (0.36; 0.65)	-
Publish Year	0.05 (-0.29; 0.39)	0.50 (0.37; 0.65)	4.1%
Mean Age	0.36 (0.05; 0.68)^a^	0.47 (0.34; 0.63)	-8.0%
Percentage female	0.28 (-0.03; 0.60)	0.47 (0.35; 0.63)	-8.0%
Sample Size	0.26 (-0.05; 0.59)	0.48 (0.36; 0.64)	-4.0%
Exercise period	0.23 (-0.08; 0.65)	0.49 (0.36; 0.65)	0%
Exercise dose	-0.51 (-0.80; -0.22)^a^	0.41 (0.28; 0.56)	-30.0%

*CrI* Credible Interval

^a^Significant influence factors

### Dose–response relationships

**Fig. 4:**
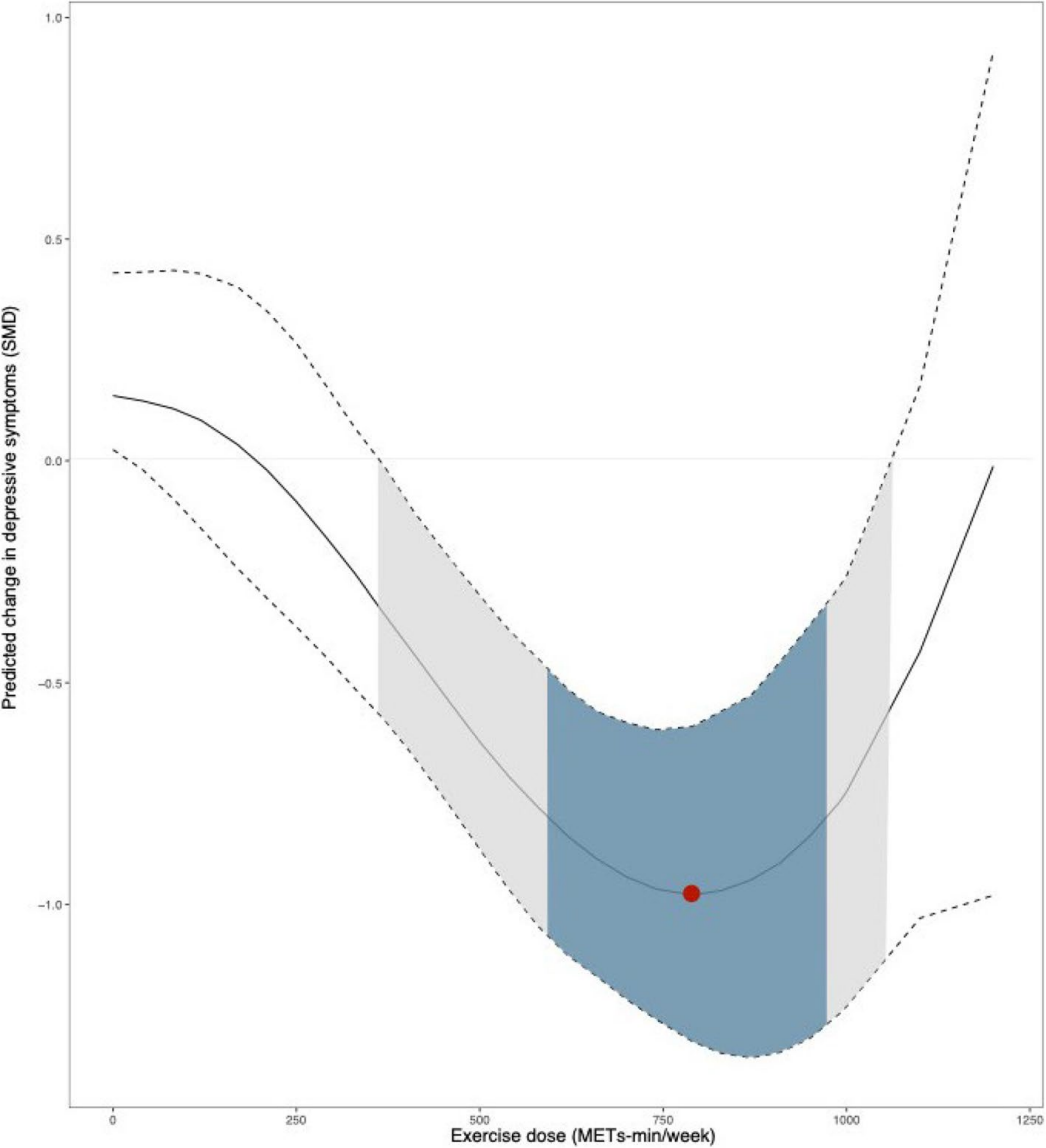
Dose–response association between total exercise dose and change in depressive symptoms in older adults. The gray shaded areas represent significantly effective, and the red points represents the optimal effect dose. The blue shaded areas represent the clinical effect

We detected U-shaped dose–response relationship between exercise dose and depressive symptoms for total exercises (Fig. 4). Predicted maximal significant response was observed at 800 METs-min/week (SMD: -0.97; 95% Crl (-1.30 to -0.63)). We observed that 350-1000METs-min/week was the dose range in which exercise appears to significantly relieve depressive symptoms in the older adults.

**Fig. 5:**
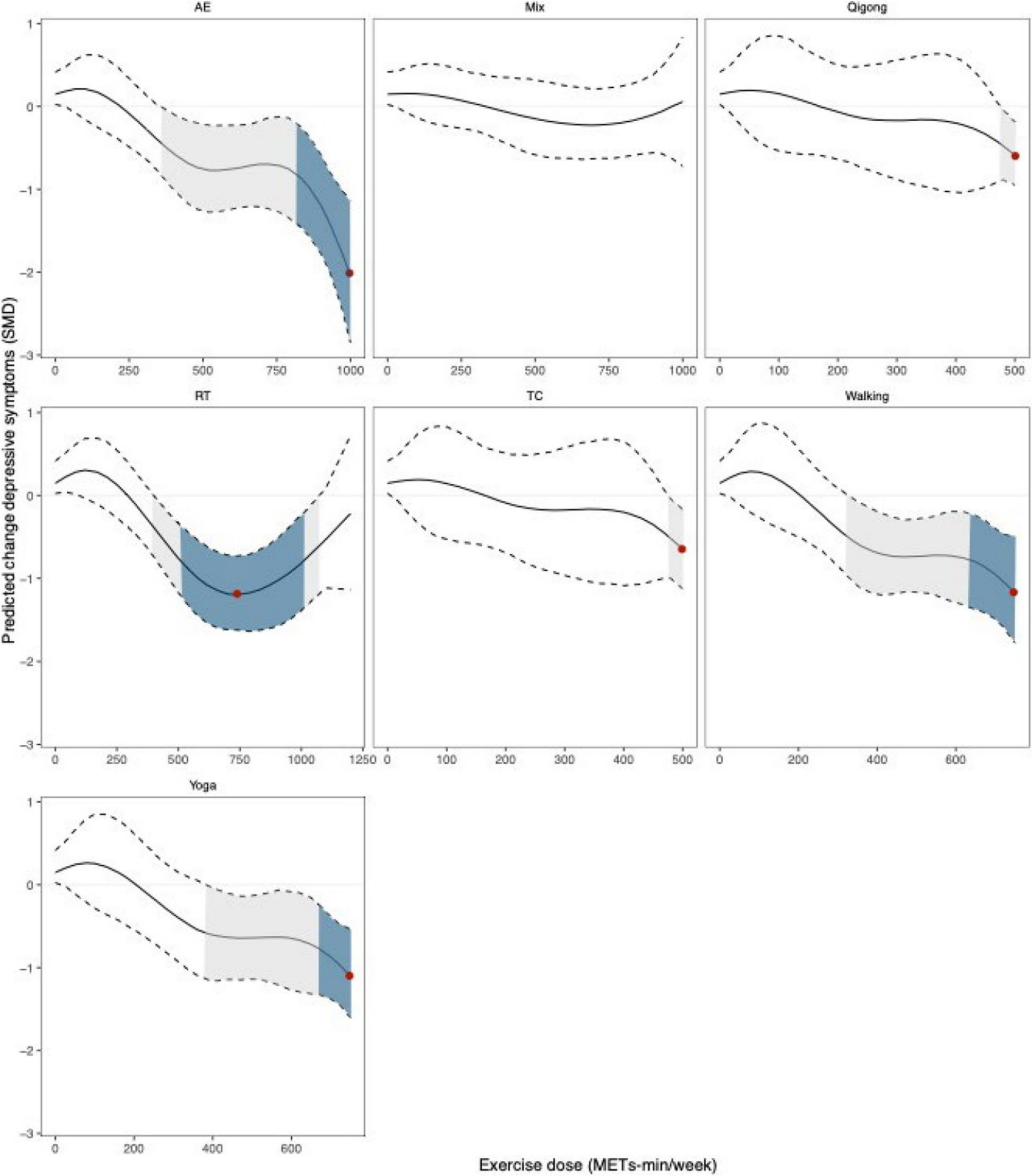
Dose–response association between dose of each exercise type and change in depressive symptoms in older adults. The gray shaded areas represent significantly effective, and the red points represents the optimal effect dose. The blue shaded areas represent the clinical effect

Different types of exercise led to different non-linear dose relationships between exercise dose and depressive symptoms in the older adults, excepted for RT and Mix (U-shaped dose–response relationship). Figure 5 showed that AE, Qigong, TC, Walking and Yoga all had a non-linear increase in the benefit of relieving depressive symptoms with the increase of exercise dose. AE presented the optimal dose at 1000METs-min/week (SMD: -2.02; 95% Crl (-2.80 to -1.17)) in this study, and the minimum effective dose was 350 METs-min/week (SMD: -0.44; 95% Crl (-0.84 to -0.25)). In addition, for Qigong and TC, the minimum effective dose was 480METs-min/week, Walking was 250 METs-min/week, and Yoga was 390 METs-min/week.

We also detected U-shaped dose–response relationship between exercise dose and depressive symptoms for RT and Mix (Fig. 5). However, Mix was not effective in alleviating depressive symptoms in older adults at the exercise dose range evaluated. For RT, the predicted maximal significant response was observed at 750 METs-min/week (SMD: -1.93; 95% Crl (-1.64 to -0.71)), and 400-1000METs-min/week was the dose range in which exercise appears to significantly relieve depressive symptoms in the older adults.

### Clinical effect

The pooled effect size equivalent to the estimated MCID for GDS-15 was large (SMD = 0.80; 95% Crl (-0.99 to -0.60) and the predicted dose of exercise (total) required to achieve this effect was 600–970 METs-min/week (Fig. 4). Corresponding to a specific exercise type, RT was 520–1000 METs-min/week. The dose of AE required to achieve this effect was a minimum of 820 METs-min/week, Walking was 650 METs-min/week, and Yoga was 680 METs-min/week. For the rest of the exercise types, there were no corresponding exercise doses to achieve clinical benefits (Fig. 5).

### Effect of age on exercise relief of depressive symptoms

**Fig. 6:**
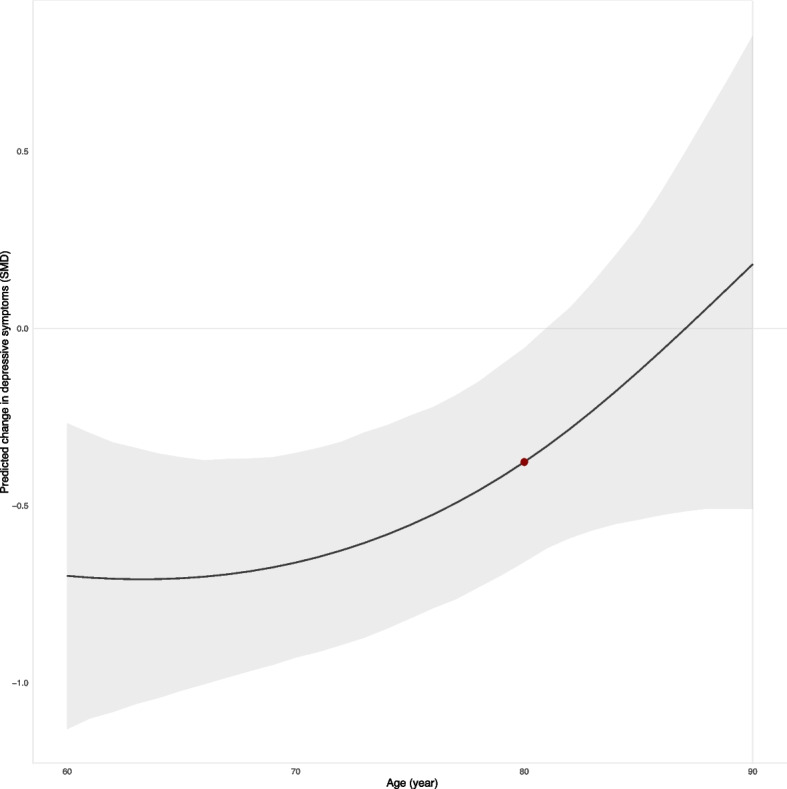
The relationship between age and the effectiveness of exercise in alleviating depressive symptoms in the older adults. The gray shaded areas represent 95% Crl, and the red points represents the inflection point where the efficacy declines at an accelerated rate (80 years old)

Since age was a significant factor in the relief of depressive symptoms by exercise in the older adults, our results using the nonlinear relationship fitted by restricted cubic splines revealed that the effect of exercise on relieving depressive symptoms in the older adults gradually decreased with age (Fig. 6), at the age of 80, the inflection point of acceleration appeared in the decline of the mitigation effect (linear slope = 0.03 for every additional year), and no significant relieved effect after age ≥ 81 years.

## Discussion

To our knowledge, this study is the first comprehensive dose–response meta-analysis of the relationship between exercise and depression in older adults with ultimately included 47 randomized controlled trials involving 2895 participants. First, our findings found that Walking, AE, Yoga, Qigong, RT, and TC all significantly alleviated depressive symptoms in older adults compared with the control group, and the effect size was moderate to higher (SMD > 0.5)[45], and Walking had the best effects. Second, this dose–response meta-analysis emphasized the in U-shaped dose–response relationship between exercise and depressive symptoms, with an effective dose range of 350 ~ 1000METs-min/week, and the optimal dose was 800METs-min/week (clinically effective: 600–970 METs-min/week). Third, except for RT and Mix, the benefits of other exercise in alleviating depressive symptoms increased nonlinearly with increasing dose. Among them, AE has the best effect at 1000METs-min/week, and it is worth nothing that Walking appears to effectively alleviate depressive symptoms at very low dose (250METs-min/week). Fourth, when considering the MCID, the minimum threshold dose for AE, Walking and Yoga was 820METs-min/week, 650METs-min/week and 680METs-min/week, respectively, and the effective dose of RT ranges from 520 ~ 1000METs-min/week, other types of exercise were not clinical effective. Fifth, early exercise will help alleviate depression, however, the decline in the effect of exercise on improving depressive symptoms will accelerate from the age of 80, and after the age of 81, exercise will not be significantly effective in alleviating depressive symptoms. Taken together, our findings provide an opportunity for future exercise guidelines aimed at improving quality of life and reducing the life burden associated with depression among older adults in this growing population.

Exercise is an effective means of alleviating depressive symptoms in older adults, and our study supports this view. Prior research has underscored the significant impact of exercise on depression, primarily attributable to the augmentation of β-endorphin release, increased availability of brain neurotransmitters (e.g., serotonin, dopamine, and norepinephrine), and heightened levels of brain-derived neurotrophic factors [46]. Moreover, exercise have been associated with enhanced self-esteem, self-assessment, and a heightened sense of achievement among individuals [47], while also displaying a positive correlation with self-efficacy [48]. Simultaneously, exercise provides opportunities for social interaction, which is crucial for the older adults who often experience feelings of loneliness and isolation. By participating in exercise groups or fitness classes, they appears to establish support networks and connect with others, which is paramount for their psychological well-being [49].

In the absence of considering exercise dosage, our research findings indicated that Walking had the most optimal effect in alleviating depressive symptoms in elderly individuals. As a form of physical activity, the inherent advantages of Walking in mitigating depressive symptoms in the older adults were previously described in the text. However, the reason for its outstanding performance in alleviating depressive symptoms may have been associated with its ease of implementation and widespread feasibility. Elderly individuals typically found it easy to initiate Walking exercises without the need for specialized equipment or high technical requirements, which contributed to increased participation in physical activity. Additionally, Walking is commonly conducted outdoors, allowing older adults to enjoy natural surroundings [50] while benefiting from exposure to sunlight and fresh air [51], thus aiding in mood enhancement. In summary, Walking, as a simple, easily implementable, and cost-effective form of exercise, demonstrated a significant impact in alleviating depressive symptoms in the older adults. This finding offers crucial insights into the prevention and treatment of depression in the older adults while emphasizing the potential for promoting Walking as a health promotion measure. Nevertheless, further research is warranted to explore the effectiveness of Walking across different age groups and health conditions, in order to comprehensively understand its applicability in diverse populations.

In the absence of considering exercise types, our research findings indicated a U-shaped relationship between exercise and the alleviation of depressive symptoms in the older adults, and our study provided statistically (350–1000 METs-min/week) and clinically (600–970 METs-min/week) meaningful dosage ranges with the optimal dose (800 METs-min/week). Firstly, the beneficial effects of moderate exercise dosages on the mitigation of depressive symptoms in the older adults may originate from physiological and psychological mechanisms. Moderate exercise appears to stimulate the release of neurotransmitters, such as dopamine and endorphins, thereby improving emotional states. However, insufficiently low exercise dosages may not be adequate to trigger these physiological responses, whereas excessively high dosages may lead to physical fatigue, increased emotional burden, and hinder the alleviation of depressive symptoms. The significance of this U-shaped relationship lies in its reminder to healthcare professionals and elderly depression patients to exercise caution when selecting exercise therapy regimens. Individual assessments and medical guidance are of paramount importance to ensure that every elderly individual appears to benefit from exercise therapy while mitigating potential risks. Furthermore, there is currently a lack of clinical studies comparing different exercise dosages concerning the physiological and psychological mechanisms in the elderly. In the future, more high-quality clinical experiments are required to validate our conclusions.

Our research findings indicated that the relationship between exercise dosage and depressive symptoms in the elderly varied depending on the type of exercise. Among the seven existing exercise types, five of them (AE, Qigong, TC, Walking, and Yoga) exhibited a non-linear increase in their efficacy in alleviating depressive symptoms with increasing exercise dosage. Furthermore, the dosage-response relationship for resistance training (RT) in mitigating depressive symptoms resembled the overall pattern of exercise (U-shaped relationship). When comparing exercise dosages across different exercise types, it became evident that only RT had an exercise dosage exceeding 1000 METs*min/week, unlike the other exercise types, which may explain this phenomenon. Previous research results have shown a significant relationship between the autonomic nervous system and depressive symptoms. For instance, heart rate variability decreased with the severity of depressive symptoms (*r* = -0.354, *p* < 0.001) [52]. Although existing research did not compare the reasons for differences in alleviating depressive symptoms between different types of exercise or exercise dosages, we gleaned from the limited studies available that resistance training does not appear to have the same consistent efficacy in improving heart rate variability as aerobic exercise [53, 54]. The results of another study indicated that, compared to a non-exercise control group, high-intensity resistance training (2 sessions/week, 7 exercises, 2–4 sets, 10–4 RM) did not significantly improve relevant indicators of the autonomic nervous system in elderly individuals [55]. However, unfortunately, this study did not establish a low-intensity resistance training control group, and thus we cannot entirely attribute the inconsistent effects on the autonomic nervous system to exercise dosage.

It is important to note that AE, rather than Walking, demonstrated its optimal efficacy in alleviating depressive symptoms in the older adults at a dosage of 1000 METs*min per week. Previous research findings have suggested that the improvement in the autonomic nervous system of the older adults increases with the rising dosage of aerobic exercise [56]. The enhancement of the autonomic nervous system contributes to the amelioration of depressive symptoms in the older adults [52], which appears to partly explain our results. Furthermore, it is noteworthy that our pooled results indicated that the maximum dosage for AE is 1000 METs*min per week. Whether the effect on alleviating depressive symptoms in the older adults continues to increase with a further increase in exercise dosage will need further research for validation. Additionally, our research results also showed that Walking was significantly effective in alleviating depressive symptoms in the older adults at dosages exceeding 250 METs*min per week. This is especially important for elderly individuals who may not be able to engage in high-intensity or high-difficulty exercises. At the same time, clinical doctors and healthcare professionals can recommend elderly individuals to commence exercise based on this minimal dosage threshold to improve their mental well-being.

This study established the use of the MCID as a meaningful metric for evaluating the relief of depressive symptoms, thus adding a level of clinical relevance to our findings. Our research results indicated that AE (≥ 820 METs-min/week), RT (520–1000 METs-min/week), Walking (≥ 650 METs-min/week), and Yoga (≥ 680 METs-min/week) all exhibited significant clinical benefits within their respective dosage ranges. This provides valuable insights to both patients and clinicians, emphasizing a practical approach to improving depressive symptoms in older adults. Furthermore, we offered a variety of clinically effective exercise types for selection, along with clear exercise dosages. Therefore, clinical doctors, healthcare professionals, or patients can make personalized choices based on individual preferences and physical conditions, leading to the most effective improvement of depressive symptoms in older adults.

We recognized that it was critical to distinguish the difference between symptom relief and a diagnosis of depression. Particularly when using the MCID as a metric, although improvement in symptoms might have reached clinical significance, it did not mean that the patient was completely free from a diagnosis of depression. This distinction was particularly important because misinterpreting improvement in symptoms as complete remission of the condition could lead to a Type I error—misjudgment of the presence of an effect when none was actually present [24]. Future studies should aim to elucidate the long-term effects of exercise on depressive disorders, distinguishing differences between symptom relief and actual changes in diagnostic status. By doing so, we could better understand the role of exercise in the broader field of depression management and ensure that treatment strategies were both effective and appropriately tailored to the needs of individual patients.

In addition, our research results evaluated the impact of age on the efficacy of exercise in alleviating depressive symptoms in older adults and indicated that individuals over the age of 81 did not experience significant relief from depressive symptoms even when engaging in exercise. Prior research results have suggested that age-related declines in central serotonin function may render older adults more susceptible to depression and may result in more frequent, severe, and treatment-resistant depressive episodes [57]. This could be a potential explanation for the findings of this study. Simultaneously, our research results hold significant implications for clinicians and patients, emphasizing that engaging in exercise as early as possible may result in more noticeable relief of depressive symptoms in older adults.

### Limitation and strength

Our study exhibited limitations. Firstly, our network meta-analysis displayed substantial heterogeneity (I^2^ = 70.8%, τ^2^ = 0.1962, *p* < 0.0001). We conducted network meta-regression to identify the sources of heterogeneity, revealing that exercise dosage and age significantly influenced the heterogeneity in this study. This is also why we delved into exploring the reasons for how exercise dosage and age affect the efficacy of exercise in alleviating depressive symptoms in older adults. Simultaneously, this heterogeneity might introduce some uncertainty to the study results. Additionally, there is the possibility of publication bias in our study (Egger test: *P* = 0.059, Supplementary 6). Because studies with negative or non-significant results are less likely to be published, our findings might overestimate the effectiveness of exercise in mitigating depressive symptoms in older adults. The study primarily focused on older adults, and the findings may not be directly applicable to other age groups. Generalizing these results to younger populations should be done with caution. While the study explored dose–response relationships, it did not extensively elucidate the pathophysiological and psychological mechanisms underlying the observed effects. Further research is needed to understand the involved physiological and psychological mechanisms.

However, despite these limitations, this study remains the first comprehensive dose–response meta-analysis regarding the relationship between exercise and depression in older adults, encompassing a substantial number of trials and participants. This adds substantial weight to the findings. Furthermore, the incorporation of the MCID as a metric for assessing clinical relevance enhances the practical significance of the results, allowing for more meaningful application in clinical settings. The identification of effective dosage ranges, optimal dosages, and minimum threshold dosages for different exercise types provides clear and actionable guidance for clinicians and patients regarding exercise interventions. Notably, the study considered age-related variations in the effectiveness of exercise on depressive symptoms, particularly the accelerated decline in efficacy after the age of 80, which is valuable for tailoring exercise recommendations to specific age groups.

## Conclusion

In conclusion, our extensive analysis of 47 randomized controlled trials involving 2895 older adults has provided significant insights: Various exercises, including Walking, AE, Yoga, Qigong, RT, and TC, effectively alleviate depressive symptoms in older adults. Furthermore, we established statistically and clinically significant threshold doses for various exercise types. Early initiation of exercise is beneficial, but its efficacy diminishes from the age of 80, and beyond 81, exercise no longer significantly alleviates depressive symptoms. These findings offer valuable insights for the development of exercise guidelines to enhance the quality of life and reduce the burden of depression in the growing population of older adults

### Supplementary Information

Supplementary Material 1.

## Data Availability

Data supporting the results of the study can be made available by emailing the first author or corresponding author.
