# Supplementary material for: Optimal dose and type of exercise to improve depressive symptoms in older adults: a systematic review and network meta-analysis
Source: BMC Geriatr. 2024 Jun 7;24:505. doi: 10.1186/s12877-024-05118-7 (PMC11157862; doi:10.1186/s12877-024-05118-7)
Supplement: Supplementary file 1 — Supplementary Material 1. [file 12877_2024_5118_MOESM1_ESM.docx]

**Supplementary**

Table of Contents:

[Supplementary 1: Search Strategy 3](#_Toc143793371)

[Database: PubMed <inception to July 15 2023> 3](#_Toc143793372)

[Database: Ovid MEDLINE(R) <1946 to July 15 2023> 5](#_Toc143793372)

[Database: Embase <1974 to July 15 2023> 6](#_Toc143793372)

[Database: PsycINFO <1806 to July 15 2023> 7](#_Toc143793372)

[Cochrane 9](#_Toc143793372)

[Database: Web of Science <1965 to July 15 2023> 1](#_Toc143793372)0

[Supplementary 2: Assessment of the transitivity 1](#_Toc143793373)2

[2.1 Publish years 1](#_Toc143793374)2

[2.2 Mean age 1](#_Toc143793375)3

[2.3 Percentage female 1](#_Toc143793376)4

[2.4 Sample size 1](#_Toc143793377)5

[2.5 Exercise period 1](#_Toc143793378)6

[2.6 Exercise dose 1](#_Toc143793379)7

[Supplementary 3: Characteristics of studies and subjects included in the review 1](#_Toc143793380)8

[Supplementary 4: Risk of Bias 3](#_Toc143793381)1

[Table 4.1 The risk of bias assessment for the individual included studies 3](#_Toc143793382)1

[Supplementary 5: Evaluation of inconsistency 3](#_Toc143793383)7

[Supplementary 6: Publication bias 3](#_Toc143793384)9

[Supplementary 7: Grading the evidence for depression symptoms of 4](#_Toc143793385)0

[7.1 Summary of study limitations of the included studies 4](#_Toc143793386)0

[7.2 Reasons for downgrading 4](#_Toc143793387)1

[7.3 CINeMA for the primary outcome “depression symptoms” 4](#_Toc143793388)3

[Supplementary 8: Assessment of Connectivity, Consistency and Transitivity in Network Meta Dose-Response Analysis 4](#_Toc143793389)8

[Supplementary 9: Non-linear functions and models fit comparison 5](#_Toc143793390)1

# Supplementary 1: Search Strategy

## Database: PubMed <inception to July 15 2023>

***Search Strategy:***

| Search number | Query |
| --- | --- |
| 27 | (((((exercise*[MeSH Terms] OR resistance training[MeSH Terms]) OR Tai Ji[MeSH Terms]) OR Qigong[MeSH Terms]) OR Yoga[MeSH Terms]) OR Exercise Movement Techniques[MeSH Terms]) OR ((aerobic exercise) OR (aquatic exercise) OR (balance training) OR (body weight support treadmill) OR (hiking) OR (high-speed resistance training) OR (multicomponent exercise program) OR (Walking) OR (power training) OR (treadmill training) OR (whole body vibration) OR (running) OR (jogging) OR (cycling) OR (walk*) OR (swim*) OR (activit*) OR (physical medicine)[Title/Abstract])) AND ((((((((randomized controlled trial[Publication Type]) OR (controlled clinical trial[Publication Type])) OR (randomized[Title/Abstract])) OR (placebo[Title/Abstract])) OR (randomly[Title/Abstract])) OR (trial[Title])) OR (clinical trials as topic[MeSH Terms])) NOT ((animals[MeSH Terms]) NOT (humans[MeSH Terms])))) AND ((Aged[MeSH Terms]) OR ((elderly) OR (older adult) OR (older people)))) AND ((Depression[MeSH Terms]) OR ((Depressive Symptoms) OR (Depressive Symptom) OR (Symptom, Depressive) OR (Emotional Depression) OR (Depression, Emotional))) |
| 26 | (Depression[MeSH Terms]) OR ((Depressive Symptoms) OR (Depressive Symptom) OR (Symptom, Depressive) OR (Emotional Depression) OR (Depression, Emotional)) |
| 25 | (Aged[MeSH Terms]) OR ((elderly) OR (older adult) OR (older people)) |
| 24 | (((((((randomized controlled trial[Publication Type]) OR (controlled clinical trial[Publication Type])) OR (randomized[Title/Abstract])) OR (placebo[Title/Abstract])) OR (randomly[Title/Abstract])) OR (trial[Title])) OR (clinical trials as topic[MeSH Terms])) NOT ((animals[MeSH Terms]) NOT (humans[MeSH Terms])) |
| 23 | ((((((randomized controlled trial[Publication Type]) OR (controlled clinical trial[Publication Type])) OR (randomized[Title/Abstract])) OR (placebo[Title/Abstract])) OR (randomly[Title/Abstract])) OR (trial[Title])) OR (clinical trials as topic[MeSH Terms]) |
| 22 | (((((exercise*[MeSH Terms] OR resistance training[MeSH Terms]) OR Tai Ji[MeSH Terms]) OR Qigong[MeSH Terms]) OR Yoga[MeSH Terms]) OR ((aerobic exercise) OR ((Exercise Movement Techniques) OR (aquatic exercise) OR (balance training) OR (body weight support treadmill) OR (hiking) OR (high-speed resistance training) OR (multicomponent exercise program) OR (Walking) OR (power training) OR (treadmill training) OR (whole body vibration) OR (running) OR (jogging) OR (cycling) OR (walk*) OR (swim*) OR (activit*) OR (physical medicine)[Title/Abstract])) |
| 21 | (animals[MeSH Terms]) NOT (humans[MeSH Terms]) |
| 20 | humans[MeSH Terms] |
| 19 | animals[MeSH Terms] |
| 18 | clinical trials as topic[MeSH Terms] |
| 17 | trial[Title] |
| 16 | randomly[Title/Abstract] |
| 15 | placebo[Title/Abstract] |
| 14 | randomized[Title/Abstract] |
| 13 | controlled clinical trial[Publication Type] |
| 12 | randomized controlled trial[Publication Type] |
| 11 | (aerobic exercise) OR (aquatic exercise) OR (balance training) OR (body weight support treadmill) OR (hiking) OR (high-speed resistance training) OR (multicomponent exercise program) OR (Walking) OR (power training) OR (treadmill training) OR (whole body vibration) OR (running) OR (jogging) OR (cycling) OR (walk*) OR (swim*) OR (activit*) OR (physical medicine)[Title/Abstract] |
| 10 | Exercise Movement Techniques[MeSH Terms] |
| 9 | Yoga[MeSH Terms] |
| 8 | Qigong[MeSH Terms] |
| 7 | Tai Ji[MeSH Terms] |
| 6 | resistance training[MeSH Terms] |
| 5 | exercise*[MeSH Terms] |
| 4 | (Depressive Symptoms) OR (Depressive Symptom) OR (Symptom, Depressive) OR (Emotional Depression) OR (Depression, Emotional) |
| 3 | Depression[MeSH Terms] |
| 2 | (elderly) OR (older adult) OR (older people) |
| 1 | Aged[MeSH Terms] |

## Database: Ovid MEDLINE(R) <1946 to July 15 2023>

***Search Strategy:***

1 Depression$.mp.

2 exp depressive disorder/

3 Aged$.mp

4 exp elderly/

5 exp older adult/

6 exp older people/

7 (aerobic exercise or aquatic exercise or balance training or body weight support treadmill or physical activity or exercise$ or walk$ or hiking or high-speed resistance training or hydrotherapy or multicomponent exercise program or multidisciplinary exercise program or Nordic Walking or Physiotherapy or pilates or power training or Qigong or resistance training or running or stretch or tai ji or cycling or treadmill training or phycial medicine or activit$ or jogging or Yoga).mp.

8 exp resistance training/

9 exp exercise$/

10 exp tai ji/

11 exp Qigong/

12 exp exercise movement techniques/

13 exp Yoga/

14 exp Virtual Reality/

15 exp hydrotherapy/

16 exp physical medicine/

17 randomized controlled trial.pt.

18 controlled clinical trial.pt.

19 randomized.ab.

20 clinical trials as topic.sh.

21 randomly.ab.

22 trial.ti.

23 exp clinical trial/

24 exp randomized controlled trials/

25 exp cross-over studies/

26 (clinic$ adj2 trial).mp.

27 (random$ adj5 control$ adj5 trial$).mp.

28 (crossover or cross-over).mp.

29 randomi$.mp.

30 (random$ adj5 (assign$ or allocat$ or assort$ or reciev$)).mp.

31 1 or 2

32 3 or 4 or 5 or 6

33 7 or 8 or 9 or 10 or 11 or 12 or 13 or 14 or 15 or 16

34 17 or 18 or 19 or 20 or 21 or 22 or 23 or 24 or 25 or 26 or 27 or 28 or 29 or 30

35 31 and 32 and 33 and 34

## Database: Embase <1974 to July 15 2023>

***Search Strategy:***

1 Depression$.mp.

2 exp depressive disorder/

3 Aged$.mp

4 exp elderly/

5 exp older adult/

6 exp older people/

7 (aerobic exercise or aquatic exercise or balance training or body weight support treadmill or physical activity or exercise$ or walk$ or hiking or high-speed resistance training or hydrotherapy or multicomponent exercise program or multidisciplinary exercise program or Nordic Walking or Physiotherapy or pilates or power training or Qigong or resistance training or running or stretch or tai ji or cycling or treadmill training or phycial medicine or activit$ or jogging or Yoga).mp.

8 exp resistance training/

9 exp exercise$/

10 exp tai ji/

11 exp Qigong/

12 exp exercise movement techniques/

13 exp Yoga/

14 exp Virtual Reality/

15 exp hydrotherapy/

16 exp physical medicine/

17 randomized controlled trial.pt.

18 controlled clinical trial.pt.

19 randomized.ab.

20 clinical trials as topic.sh.

21 randomly.ab.

22 trial.ti.

23 exp clinical trial/

24 exp randomized controlled trials/

25 exp cross-over studies/

26 (clinic$ adj2 trial).mp.

27 (random$ adj5 control$ adj5 trial$).mp.

28 (crossover or cross-over).mp.

29 randomi$.mp.

30 (random$ adj5 (assign$ or allocat$ or assort$ or reciev$)).mp.

31 1 or 2

32 3 or 4 or 5 or 6

33 7 or 8 or 9 or 10 or 11 or 12 or 13 or 14 or 15 or 16

34 17 or 18 or 19 or 20 or 21 or 22 or 23 or 24 or 25 or 26 or 27 or 28 or 29 or 30

35 31 and 32 and 33 and 34

## Database: PsycINFO <1806 to July 15 2023>

***Search Strategy:***

| S1 | Depression* | APA PsycInfo® |
| --- | --- | --- |
| S2 | mainsubject(depressive disorder) | APA PsycInfo® |
| S3 | Aged* | APA PsycInfo® |
| S4 | mainsubject(elderly) | APA PsycInfo® |
| S5 | mainsubject(older adult) | APA PsycInfo® |
| S6 | mainsubject(older people) | APA PsycInfo® |
| S7 | su((aerobic exercise or aquatic exercise or balance training or body weight support treadmill or Dance Therapy or exercise$ or running or jogging or high-speed resistance training or hydrotherapy or multicomponent exercise program or multidisciplinary exercise program or Nordic Walking or Physiotherapy or pilates or power training or Qigong or resistance training or cycling or stretch or tai ji or phycial medicine or treadmill training or walking or Virtual Reality or whole body vibration or Yoga)) | APA PsycInfo® |
| S8 | su(exercise$) | APA PsycInfo® |
| S9 | su(physical activity) | APA PsycInfo® |
| S10 | ab(randomized) | APA PsycInfo® |
| S11 | ab(randomly) | APA PsycInfo® |
| S12 | ti(trial) | APA PsycInfo® |
| S13 | ab(clinical trial) | APA PsycInfo® |
| S14 | ab(randomized controlled trials) | APA PsycInfo® |
| S15 | ab(cross-over studies) | APA PsycInfo® |
| S16 | ab(crossover studies) | APA PsycInfo® |
| S17 | ab(randomi*) | APA PsycInfo® |
| S18 | su(animals) | APA PsycInfo® |
| S19 | S1 OR S2 | APA PsycInfo® These databases are searched for part of your query. |
| S20 | S3 OR S4 OR "S5" OR "S6" | APA PsycInfo® These databases are searched for part of your query. |
| S21 | S7 OR S8 OR "S9" | APA PsycInfo® These databases are searched for part of your query. |
| S22 | S10 OR S11 OR S12 OR "S13" OR "S14" OR "S15" OR "S16" OR S17 OR S18 | APA PsycInfo® These databases are searched for part of your query. |
| S23 | S19 AND S20 | APA PsycInfo® These databases are searched for part of your query. |
| S24 | S21 AND S22 | APA PsycInfo® These databases are searched for part of your query. |
| S25 | S23 NOT S24 | APA PsycInfo® These databases are searched for part of your query. |

## Cochrane

#1 MeSH descriptor: [depressive disorder] explode all trees

#2 MeSH descriptor: [Aged] explode all trees

#3 (aerobic exercise or aquatic exercise or balance training or body weight support treadmill or Dance Therapy or exercise* or Walking or gait training or high-speed resistance training or hydrotherapy or multicomponent exercise program or multidisciplinary exercise program or Nordic Walking or Physiotherapy or pilates or power training or Qigong or resistance training or Robotic-assisted gait training or stretch or tai ji or Running or treadmill training or jogging or Virtual Reality or whole body vibration or Yoga) in Trials (Word variations have been searched)

#4 MeSH descriptor: [resistance training] explode all trees

#5 MeSH descriptor: [exercise] explode all trees

#6 MeSH descriptor: [tai ji] explode all trees

#7 MeSH descriptor: [Qigong] explode all trees

#8 MeSH descriptor: [Exercise Movement Technique] explode all trees

#9 MeSH descriptor: [Yoga] explode all trees

#10 MeSH descriptor: [Virtual Reality] explode all trees

#11 MeSH descriptor: [hydrotherapy] explode all trees

#12 MeSH descriptor: [Dance Therapy] explode all trees

#13 MeSH descriptor: [Exercise Movement Techniques] explode all trees

#14 #3 or #4 or #5 or #6 or #7 or #8 or #9 or #10 or #11 or #12 or #13

#15 #1 and #2 and #14

## Database: Web of Science <1965 to July 15 2023>

| # 14 | #13 AND #11 AND #1 AND #2  Indexes=SCI-EXPANDED, SSCI, A&HCI, CPCI-S, CPCI-SSH, BKCI-S, BKCI-SSH, ESCI, CCR-EXPANDED, IC Timespan=All years |  |  |
| --- | --- | --- | --- |
| # 13 | #11 OR #10 OR #9 OR #8 OR #7 OR #6 OR #5 OR #4 OR #3  Indexes=SCI-EXPANDED, SSCI, A&HCI, CPCI-S, CPCI-SSH, BKCI-S, BKCI-SSH, ESCI, CCR-EXPANDED, IC Timespan=All years |  |  |
| # 12 | TOPIC: ((“randomized controlled trial*” or “controlled clinical trial” or “random*” or “clinical trial*” or randomly or trial or “clinical trial” or “randomized controlled trial*” or “cross-over studies” or clinic*) )  Indexes=SCI-EXPANDED, SSCI, A&HCI, CPCI-S, CPCI-SSH, BKCI-S, BKCI-SSH, ESCI, CCR-EXPANDED, IC Timespan=All years |  |  |
| # 11 | TOPIC: ((Yoga or “Muscle Stretching Exercises”) )  Indexes=SCI-EXPANDED, SSCI, A&HCI, CPCI-S, CPCI-SSH, BKCI-S, BKCI-SSH, ESCI, CCR-EXPANDED, IC Timespan=All years |  |  |
| # 10 | TOPIC: ((“Dance Therapy” or “Therapy, Dance” or “Dance Therapies” or “Therapies, Dance”) )  Indexes=SCI-EXPANDED, SSCI, A&HCI, CPCI-S, CPCI-SSH, BKCI-S, BKCI-SSH, ESCI, CCR-EXPANDED, IC Timespan=All years |  |  |
| # 9 | TOPIC: ((hydrotherapy or Hydrotherapies or “Whirlpool Baths” or “Bath, Whirlpool” or “Baths, Whirlpool” or “Whirlpool Bath”) )  Indexes=SCI-EXPANDED, SSCI, A&HCI, CPCI-S, CPCI-SSH, BKCI-S, BKCI-SSH, ESCI, CCR-EXPANDED, IC Timespan=All years |  |  |
| # 8 | TOPIC: (("Virtual Reality" or "Reality, Virtual" or "Virtual Reality, Educational" or "Educational Virtual Realities" or "Educational Virtual Reality" or "Reality, Educational Virtual" or "Virtual Realities, Educational" or "Virtual Reality, Instructional" or "Instructional Virtual Realities" or "Instructional Virtual Reality" or "Realities, Instructional Virtual" or "Reality, Instructional Virtual" or "Virtual Realities, Instructional") )  Indexes=SCI-EXPANDED, SSCI, A&HCI, CPCI-S, CPCI-SSH, BKCI-S, BKCI-SSH, ESCI, CCR-EXPANDED, IC Timespan=All years |  |  |
| # 7 | TOPIC: (("Exercise Movement Technics" or "Movement Techniques, Exercise" or "Pilates-Based Exercises" or "Exercises, Pilates-Based" or "Pilates Based Exercises" or "Pilates Training" or "Training, Pilates"）)  Indexes=SCI-EXPANDED, SSCI, A&HCI, CPCI-S, CPCI-SSH, BKCI-S, BKCI-SSH, ESCI, CCR-EXPANDED, IC Timespan=All years |  |  |
| # 6 | TOPIC: (“Tai-ji” or “Tai Chi” or “Chi, Tai” or “Tai Ji Quan” or “Ji Quan, Tai” or “Quan, Tai Ji” or Taiji or Taijiquan or “T'ai Chi” or “Tai Chi Chuan” Qigong or “Qi Gong” or “Ch'i Kung”)  Indexes=SCI-EXPANDED, SSCI, A&HCI, CPCI-S, CPCI-SSH, BKCI-S, BKCI-SSH, ESCI, CCR-EXPANDED, IC Timespan=All years |  |  |
| # 5 | TOPIC: (Exercise* or “Exercise Program, Weight-Bearing” or “Exercise Programs, Weight-Bearing” or “Weight Bearing Exercise Program” or “Weight-Bearing Exercise Programs” Exercise* or “Physical Activity” or “Activities, Physical” or “Activity, Physical” or “Physical Activities” or “Exercise, Physical” or “Exercises, Physical” or “Physical Exercise” or “Physical Exercises” or “Exercise, Isometric” or “Exercises, Isometric” or “Isometric Exercises” or “Isometric Exercise” or “Exercise, Aerobic” or “Aerobic Exercise” or “Aerobic Exercises” or “Exercises, Aerobic” or “Exercise Training” or “Exercise Trainings” or “Training, Exercise” or “Trainings, Exercise”)  Indexes=SCI-EXPANDED, SSCI, A&HCI, CPCI-S, CPCI-SSH, BKCI-S, BKCI-SSH, ESCI, CCR-EXPANDED, IC Timespan=All years |  |  |
| # 4 | TOPIC: ("Resistance training” or “Training, Resistance” or “Strength Training” or “Training, Strength” or “Weight-Lifting Strengthening Program” or “Strengthening Program, Weight-Lifting” or “Strengthening Programs, Weight-Lifting” or “Weight Lifting Strengthening Program” or “Weight-Lifting Strengthening Programs” or “Weight-Lifting Exercise Program” or “Exercise Program, Weight-Lifting” or “Exercise Programs, Weight-Lifting” or “Weight Lifting Exercise Program” or “Weight-Lifting Exercise Programs” or “Weight-Bearing Strengthening Program” or “Strengthening Program, Weight-Bearing” or “Strengthening Programs, Weight-Bearing” or “Weight Bearing Strengthening Program” or “Weight-Bearing Strengthening Programs” or “Weight-Bearing Exercise Program”)  Indexes=SCI-EXPANDED, SSCI, A&HCI, CPCI-S, CPCI-SSH, BKCI-S, BKCI-SSH, ESCI, CCR-EXPANDED, IC Timespan=All years |  |  |
| # 3 | TOPIC: (“aerobic exercise” or “aquatic exercise” or “balance training” or “body weight support treadmill” or “Dance Therapy or exercise*” or “jogging” or “gait training” or “high-speed resistance training” or “hydrotherapy” or “multicomponent exercise program” or “multidisciplinary exercise program” or “Nordic Walking” or “Physiotherapy” or pilates or “power training” or Qigong or “resistance training” or “Robotic-assisted gait training” or stretch or “tai ji” or Running or “treadmill training” or “walking” or “Virtual Reality” or “whole body vibration” or Yoga)  Indexes=SCI-EXPANDED, SSCI, A&HCI, CPCI-S, CPCI-SSH, BKCI-S, BKCI-SSH, ESCI, CCR-EXPANDED, IC Timespan=All years |  |  |
| # 2 | TOPIC: ("Aged" or "elderly")  Indexes=SCI-EXPANDED, SSCI, A&HCI, CPCI-S, CPCI-SSH, BKCI-S, BKCI-SSH, ESCI, CCR-EXPANDED, IC Timespan=All years |  |  |
| # 1 | TOPIC: ("depression" or "depressive disorder" or "Disorder, Depressive" or "Disorders, Depressive" or "Neurosis, Depressive" or "Depressive Neurosis" or "Endogenous Depression" or "Depressive Syndrome" or "Unipolar Depression" or "Depression, Unipolar" or "Unipolar Depressions")  Indexes=SCI-EXPANDED, SSCI, A&HCI, CPCI-S, CPCI-SSH, BKCI-S, BKCI-SSH, ESCI, CCR-EXPANDED, IC Timespan=All years |  |  |

# Supplementary 2: Assessment of the transitivity

Different clinical trials need to ensure that their baseline levels are consistent. If the baseline levels are inconsistent, the results cannot be transitive. Therefore, the transitivity assumption was evaluated by comparing the distribution of potential effect modifiers (publication year, sample size, mean age, percentage male, years of diagnosis, and disease grade) across studies grouped before analyzing the results, and we use the R ggplot2 package to draw boxplots between the above potential influencing factors and various types of exercise.

## 2.1 Publish years

We checked the publication year distribution of the included studies. The range is from 1990 to 2023, with a median of 2011. The results of one-way ANOVA showed that there was no statistical difference in the years of publication between the types of exercise (F = 0.548, P = 0.796).

******

**Figure 2.1:** Boxplot for distribution of publication year. *AE* Aerobic Exercise, *CON* Control group, *Mix* Mixed exercise, RT Resistance Training, *TC* Tai Chi.

## 2.2 Mean age

We checked the mean age distribution of the included study participants. The range is from 59.6 to 87.9, with a median of 72.58. The results of one-way ANOVA showed that there was no statistical difference in the mean age between the types of exercise (F = 1.458, P = 0.189).

**Figure 2.2:** Boxplot for distribution of mean age. *AE* Aerobic Exercise, *CON* Control group, *Mix* Mixed exercise, RT Resistance Training, *TC* Tai Chi.

## 2.3 Percentage female

We checked the percentage female distribution of the included study participants. The range is from 0 to 100%, with a median of 69.23. The results of one-way ANOVA showed that there was no statistical difference in the percentage female between the types of exercise (F = 1.201, P = 0.310).

******

**Figure 2.3:** Boxplot for distribution of percentage female. *AE* Aerobic Exercise, *CON* Control group, *Mix* Mixed exercise, RT Resistance Training, *TC* Tai Chi.

## 2.4 Sample size

We checked the sample size distribution of the included studies each arm. The range is from 5 to 83, with a median of 23. The results of one-way ANOVA showed that there was no statistical difference in the percentage female between the types of exercise (F = 1.358, P = 0.230).

**Figure 2.4:** Boxplot for distribution of sample size. *AE* Aerobic Exercise, *CON* Control group, *Mix* Mixed exercise, RT Resistance Training, *TC* Tai Chi.

## 2.5 Exercise period

We checked the exercise period distribution of the included studies. The range is from 2 to 26, with a median of 12. The results of one-way ANOVA showed that there was statistical difference in the exercise period between the types of exercise (F = 0.869, P = 0.523).

**Figure 2.5:** Boxplot for distribution of exercise period. *AE* Aerobic Exercise, *Mix* Mixed exercise, RT Resistance Training, *TC* Tai Chi.

## 2.6 Exercise dose

We checked the exercise dose (exercise intensity*frequency*per session time) distribution of the included studies. The range is from 180 to 1179 Metabolic Equivalent of Energy-min/week (MET-min/week), with a median of 487.5. The results of one-way ANOVA showed that there was statistical difference in the exercise period between the types of exercise (F = 0.962, P = 0.459).

**Figure 2.6:** Boxplot for distribution of exercise dose. *AE* Aerobic Exercise, *Mix* Mixed exercise, RT Resistance Training, *TC* Tai Chi.

# Supplementary 3: Characteristics of studies and subjects included in the review

| **Study** | **Age  (mean ± sd)** | **Sample size  (F/M)** | **Depression diagnosis** | **Cut off** | **Exercise type** | **P** | **F** | **T** | **Outcome** | **Adherence** |
| --- | --- | --- | --- | --- | --- | --- | --- | --- | --- | --- |
| Antunes, et al. (2005) ^1^ | AE: 68.08±5.49 CON: 65.86±3.80 | AE: 23(0/23) CON: 23(0/23) | AE: 7.95±5.66 CON: 7.73±4.56 | >10 | AE: ergometric cycle, working at a heart rate corresponding to ventilatory threshold (VT-1) intensity; CON: placebo | 24 | 3 | 20-60 | The 30-item Geriatric Depression Scale | NA |
| Bernard, et al. (2015) ^2^ | Walking: 65.46±4.37 CON: 65.5±4.03 | Walking: 61(61/0) CON: 60(60/0) | Walking: 12.31±9.12 CON: 11.32±7.65 | >13 | Walking: moderate (75% HR) supervised and home-based; CON: waiting list | 24 | 3 | 40 | Beck Depression Inventory | 50% |
| Boström, et al. (2016) ^3^ | Mix: 84.4±6.2 CON: 85.9±7.8 | Mix: 93 (70/23) CON: 93 (71/22) | > 5 | >5 | Mix: lower limb strength, balance, and mobility training; CON: usual care | 12 | 3 | 45 | The 15-item Geriatric Depression Scale | 73% |
| Bouaziz, et al. (2019) ^4^ | CON: 74.3±3.4 AE: 72.9±2.5 | CON: 30(23/7) AE: 30(21/9) | CON: 1.99±1.6 AE: 2.1±2.1 | >4 | CON: maintain their current sedentary lifestyle; AE: 4-minute cycling at the preintervention VT1 workload (called “BASE”) and 1-minute cycling at 40% of the preintervention VT1 workload (called “RECOVERY”) | 9.5 | 2 | 30 | Goldberg Anxiety and Depression Scale-Depression | 94.70% |
| Brenes, et al. (2007) ^5^ | Mix: 73.5±7.8 CON: 73.9±5.8 | Mix: 14(9/5) CON: 12(6/6) | Mix: 12.7±3.4 CON: 9.5±3.7 | >8 | Mix: aerobic and resistance training; CON: Usual care | 16 | 3 | 60 | The 17-item Hamilton Depression Rating Scale | NA |
| Brown, et al. (2009) ^6^ | Mix: 79.5±5.9 AE: 81.5±6.9 CON: 78.1±6.4 | Mix: 82 AE: 34 CON: 38 | Mix: 5.4±3.9 AE: 8.4±5.4 CON: 6.3±4.2 | >6 | Mix: specific resistance training exercises, balance (both static and dynamic) training exercises, and activities for challenging hand–eye and foot–eye co-ordination and flexibility; AE: minimal-intensity exercise CON: No-exercise | 24 | 2 | 60 | The 20-item Geriatric Depression Scale | NA |
| Chen, et al. (2009) ^7^ | 69.20±6.23 | Yoga: 62 CON: 66 | Qigong: 6.58±7.57 CON: 5.02±5.52 | >6 | Yoga: hatha yoga; CON: wait-list | 24 | 3 | 70 | Taiwanese Depression Questionnaire | NA |
| Cheng, et al. (2012) ^8^ | Tai chi: 81.0±7.7 CON: 82.5±7.1 | Tai chi: 12(6/6) CON: 12(9/3) | Tai chi: 9.25±2.14 CON: 9.08±2.11 | >5 | Tai chi: 12-form Yang style; CON: placebo | 12 | 3 | 60 | The 15-item Geriatric Depression Scale | NA |
| Chin A Paw, et al. (2004) ^9^ | RT: 81.0±5.8 Mix1: 82.1±4.9 Mix2: 80.9±6.3 CON: 81.3±4.4 | RT: 41(30/11) Mix1: 48(38/10) Mix2: 49(41/8) CON: 35(29/6) | RT: 6.0±3.4 Mix1: 5.6±4.1 Mix2: 6.1±4.3 CON: 5.2±3.6 | >10 | RT: 2 sets of 8–12 repetitions were possible; Mix1: Walking, skills training in game-like and cooperative activities; Mix2: a complete strength training session and once weekly a complete functional training session; CON: "placebo" intervention | 24 | 2 | 45~60 | The 30-item Geriatric Depression Scale | NA |
| Choi, et al. (2018) ^10^ | Yoga: 77.6±5.69 CON: 78.8±5.83 | Yoga: 33(30/3) CON: 30(29/1) | Yoga: 5.88±2.97 CON: 4.57±3.33 | >5 | Yoga: is based on the “sitting yoga program” for wheel-chair seated nursing home residents, increase the exercise intensity to 10~14 on the RPE; CON: usual care | 12 | 4 | 30~40 | The 15-item Geriatric Depression Scale Short Form Korean version | NA |
| Chou, et al. (2004) ^11^ | 72.6±4.2 | 14(7/7) | Tai chi: 32±9.9 CON: 32.7±8.7 | >15 | Tai chi:18-form of Yang’s style; CON: usual care | 12 | 3 | 45 | the Chinese version of the Center for Epidemiological Studies Depression Scale | NA |
| Clegg, et al. (2014) ^12^ | Mix: 79.4±7.9 CON: 78.0±10.5 | Mix: 45(33/12) CON: 39(27/12) | Mix: 3.8±2.7 CON: 5.0±3.2 | >5 | Mix: strength, balance, aerobic exercise; CON: usual care | 12 | 5 | 45 | The 15-item Geriatric Depression Scale | 46% |
| de Lima, et al. (2019) ^13^ | CON: 67.2 ± 5.2 RT: 66.2 ± 5.5 | CON: 16 RT: 17 | CON: 18.7 ± 5.4 RT: 17.9±8 | >8 | CON: usual care; RT:8‐12 repetitions of each of the following exercises: bench press, deadlift, unilateral rowing, standing calf raise, and abdominal reverse crunch. | 20 | 2 | 30~40 | The 17-item Hamilton Depression Rating Scale | NA |
| Emery, et al. (1990) ^14^ | 72±6 | 48(40/8) | Mix: 18.9±11.5 CON: 11.7±13.6 | >15 | Mix: 70% HRmax, stretching exercises, aerobic exercise, rhythmic muscle strengthening exercises; CON: social activity | 12 | 3 | 60 | Center for Epidemiological Studies Depression Scale | NA |
| Eyre, et al. (2017) ^15^ | Yoga: 68.1±8.7 CON: 67.6±8 | Yoga: 38(25/13) CON: 41(27/14) | Yoga: 7.7±6.57 CON: 6.51±5.28 | >5 | Yoga:60-minute KY class per week; CON: health education | 12 | 1 | 60 | The 15-item Geriatric Depression Scale | NA |
| Fakhari (2017) ^16^ | Tai chi: 69.19 ± 5.48 CON: 69.34 ± 5.03 | Tai chi: 27(13/14) CON: 29(17/12) | Tai chi: 16.18 ± 8.27 CON: 18.55 ± 9.2 | >13 | Tai chi: The ten step Tai Chi; CON: only their activity of daily living | 12 | 3 | 20~25 | Beck Depression Inventory II | NA |
| Gary, et al. (2004) ^17^ | Walking: 67±11 CON: 69±11 | Walking: 16(16/0) CON: 16(16/0) | Walking: 6±4 CON: 5±3 | >5 | Walking: walking at 60% intensity; CON: home visits | 12 | 3 | 30 | The 15-item Geriatric Depression Scale | NA |
| Gusi, et al. (2008) ^18^ | Walking: 71±5 CON: 74±6 | Walking: 55(55/0) CON: 51(51/0) | Walking: 2.3±2.5 CON: 2.6±2.5 | >5 | Walking: Each session consisted of walking alternating with specific exercises; CON: best care in general practice | 24 | 3 | 50 | The 15-item Geriatric Depression Scale | NA |
| Hsu, et al. (2016) ^19^ | Tai chi: 80.73±9.68 CON: 81.77±6.32 | Tai chi: 30(19/11) CON: 30(19/11) | Tai chi: 4.40±3.41 CON: 4.27±4.76 | >5 | Tai chi: Simplified Tai-Chi Exercise Program (STEP) developed by Chen; CON: usual standard care | 26 | 3 | 40 | Geriatric Depression Scale-Short Form (Chinese version) | 85.30% |
| Huang, et al. (2015) ^20^ | AE: 76.42±5.31 CON: 75.85±6.56 | AE: 19(11/8) CON: 20(11/9) | AE: 8.63±3.56 CON: 7.20±2.19 | >5 | CON: no intervention; AE: The intensity of the exercise was moderate | 12 | 3 | 50 | The 15-item Geriatric Depression Scale | NA |
| Ibrahim, et al. (2023) ^21^ | Moderate Walking: 62.6±5.01  low Walking: 62.5±4.67 CON: 62.7±4.3 | Moderate Walking: 24 (13/11)  low Walking: 24 (12/12) CON: 24 (16/8) | Moderate Walking: 17.17±1.58  low Walking: 17.92±2.32 CON: 17.25±1.45 | >8 | Moderate Walking: walking on treadmill, 50-70% HRmax;  low Walking: walking on treadmill, 40-50% HRmax; CON: usual care | 10 | 4 | 40 | The 17-item Hamilton Depression Rating Scale | NA |
| Kim, et al. (2019) ^22^ | RT: 76.10±3.85 CON: 76.40±3.27 | RT: 11(11/0) CON: 10(10/0) | RT: 5.0±2.49 CON: 5.2±2.2 | >5 | RT: RPE 9–13 (light-little hard) of muscle strength exercise; CON: usual-care control group | 24 | 3 | 50~80 | The 15-item Geriatric Depression Scale Short Form Korean version | NA |
| Kohut, et al. (2005) ^23^ | AE: 73.07±5.59 CON: 70.25±5.57 | AE: 14(7/7) CON: 13(7/6) | AE: 3.8±2 CON: 2.8±3.1 | >5 | AE: initially 40–60%HRR, gradually 65–75%HRR; CON: no intervention | 40 | 3 | 20~30 | The 15-item Geriatric Depression Scale | NA |
| Lin, et al. (2007) ^24^ | ≥65 | CON: 50 Mix: 50 | CON: 9.2±3.2 Mix: 9.7±3.4 | >5 | Mix: stretching, muscle strengthening, and balance training;  CON: usual care | 16 | 3 | 30~40 | The 15-item Geriatric Depression Scale | NA |
| Lincoln, et al. (2011) ^25^ | RT: 66.0±7.9 CON: 66.6±7.4 | RT: 29(20/9) CON: 29(17/12) | RT: 11.5±7.5 CON: 11.1±7.4 | >10 | RT: high-intensity progressive resistance exercise training; CON: placebo | 16 | 3 | 45 | The 30-item Geriatric Depression Scale | NA |
| Lok, et al. (2017) ^26^ | ＞65 | AE: 40(17/23) CON: 40(19/21) | AE: 18±2.62 CON: 19±1.37 | >13 | AE: rhythmic exercises, and free Walking time; CON: no planned implementation | 10 | 4 | 60 | Beck Depression Inventory | NA |
| McNeil, et al. (1991) ^27^ | 72.5±6.9 | Walking:10 CON:10 | Walking: 16.6±3.1 CON: 15.2±2.4 | >13 | Walking: walked outside near their residence; CON: Wait-list | 6 | 3 | 20~40 | Beck Depression Inventory | NA |
| Moraes, et al. (2020) ^28^ | AE: 70.88±5.94 RT: 72.89±7.06 Mix: 69.28±5.28 | AE: 9 (8/1) RT: 9(8/1) Mix: 7(5/2) | AE: 14.33±2.82 RT: 13.44±3.46 Mix: 14.57±1.81 | >13 | AE: stationary bikes or treadmills, 60% of the VO2max; RT: performed exercises for the major muscle groups, namely, 3 sets, 8-12 repetitions, 70% 1RM; Mix: low intensity aerobic and resistance training | 12 | 2 | 30 | The 17-item Hamilton Depression Rating Scale | NA |
| Netz, et al. (1994) ^29^ | Mix: 64.3±6.3 CON: 69.5±9.6 | Mix:8 CON:9 | Mix: 12.87±1.96 CON: 6.62±5.15 | >10 | Mix: included calisthenics and rhythmical movement involving large muscle movements, light; CON: discussing the news | 8 | 3 | 45 | The 30-item Geriatric Depression Scale | NA |
| Ramanathan, et al. (2017) ^30^ | Yoga: 68.90±7.55 CON: 68.20±8.78 | Yoga: 20(20/0) CON: 20(20/0) | Yoga: 19.5±4 CON: 17.5±5.75 | >8 | Yoga: Hatha; CON: wait-list | 12 | 2 | 60 | Hamilton Depression Rating Scale | NA |
| Roswiyani, et al. (2020) ^31^ | Qigong: 71.90±8.57 CON: 74.31±9.57 | Qigong: 67(47/20) CON: 65(43/22) | Qigong: 9.78±8.21 CON: 10.48±10.49 | >13 | Qigong: qi gong exercises;  CON: regular daily activities, not receive any therapeutic intervention | 8 | 2 | 90 | Beck Depression Inventory II | 61.20% |
| Sahin, et al. (2018) ^32^ | High RT: 84.18 ± 6.85 Low RT: 84.50 ± 4.81 CON: 85.37 ± 4.70 | High RT: 20(11/9) Low RT: 20(12/8) CON: 20(10/10) | High RT: 5 ± 3.22 Low RT: 3.18 ±2.25 CON: 3.43 ± 2.55 | >5 | High RT: 70% 1RM, one set of 6–10 repetitions with 6–8 s for each repetition; Low RT: 40% 1RM, one set of 6–10 repetitions with 6–8 s for each repetition; CON: usual daily routine | 8 | 3 | 40 | The 15-item Geriatric Depression Scale | NA |
| Shahidi, et al. (2011) ^33^ | Yoga: 65.5±4.8 AE: 65.7±4.2 CON: 68.4±6.3 | Yoga: 20(20/0) AE: 20(20/0) CON: 20(20/0) | Yoga: 16±5.3 AE: 15.3±5.4 CON: 15.2±3.9 | >10 | Yoga: laughter yoga; AE: jogging and stretching; CON: wait-list | 2 | 5 | 30 | The 30-item Geriatric Depression Scale | NA |
| Sims, et al. (2006) ^34^ | RT: 75.25±5.78 CON: 74.30±5.72 | RT: 14(12/2) CON: 18(9/9) | RT: 12.64±3.61 CON: 12.22±3.51 | >10 | RT: 3 sets, 80% 1RM,8-10 repetitions; CON: not receive any therapeutic intervention | 10 | 3 | 60 | The 30-item Geriatric Depression Scale | 60% |
| Sims, et al. (2009) ^35^ | RT: 67.95±14.76 CON: 66.27±16.01 | RT: 23(9/14) CON: 22(9/13) | RT: 15.43±7.49 CON: 23.27±8.86 | >15 | RT: moderate intensity (three sets of eight/ten repetitions, at a resistance of 80% of 1-RM) strengthening exercises; CON: usual care | 10 | 2 | 60 | Centre for Epidemiologic Studies for Depression scale | 75% |
| Singh, et al. (1997) ^36^ | RT: 70±1.5 CON: 72±2 | RT: 17(12/5) CON: 15(8/7) | RT: 21.3±1.8 CON: 18.3±1.7 | >13 | RT: 80% 1RM, 3 sets of 8 repetitions on each machine; CON: interactive health education program of lectures and videos | 10 | 3 | 50 | Beck Depression Inventory | NA |
| Singh, et al. (2005) ^37^ | High RT: 69±5 Low RT: 70±7 CON: 69±7 | High RT: 20(11/9) Low RT: 20(12/8) CON: 20(10/10) | High RT: 18±4.5 Low RT: 19.5±5.3 CON: 19.7±3.9 | >8 | High RT: 80% 1RM,3 sets of 8 repetitions; Low RT: 20% 1RM, 3 sets of 8 repetitions; CON: usual care | 8 | 3 | 60 | The 17-item Hamilton Depression Rating Scale | NA |
| Song, et al. (2019) ^38^ | AE: 76.22±5.76 CON: 7533±6.78 | AE: 60(48/12) CON: 60(42/18) | AE: 5.33±3.48 CON: 5.67±3.7 | >10 | AE: performed the stepping exercise in multiple bouts of at least 10min each; CON: health education programme eight bi-weekly educational classes (45 min/each session) | 16 | 3 | 60 | The 30-item Geriatric Depression Scale | 73.10% |
| Tsang, et al. (2003) ^39^ | Qigong: 72.93±9.53 CON: 76.27±8.40 | Qigong: 24(15/9) CON: 26(9/17) | Qigong: 7.39±3.91 CON: 6.05±3.4 | >10 | Qigong: The Eight Section Brocades; CON: traditional remedial rehabilitation activities | 12 | 2 | 60 | The 30-item Geriatric Depression Scale | NA |
| Tsang, et al. (2006) ^40^ | CON: 82.74±6.83 Qigong: 82.11±7.19 | CON: 34(28/6) Qigong: 48(38/10) | CON: 6.5±1.42 Qigong: 5.17±2.75 | >5 | Qigong: Baduanjin; CON: newspaper reading | 16 | 3 | 30~45 | The 15-item Geriatric Depression Scale Short Form Chinese version | NA |
| Tsang, et al. (2013) ^41^ | Qigong: 80±7 CON: 81±8 | Qigong: 21(16/5) CON: 17(10/7) | Qigong: 9.71±3.3 CON: 8.71±3.69 | >5 | Qigong: Eight-Section Brocades protocol; CON: newspaper reading and discussion program | 12 | 3 | 45 | The 15-item Geriatric Depression Scale | NA |
| Vahlberg, et al. (2017) ^42^ | Mix: 72.6±5.5 CON: 73.7±5.3 | Mix: 34(7/27) CON: 33(9/24) | Mix: 5.6±4.7 CON: 6.4±5.0 | >6 | Mix: strength, balance exercise; CON: regular activities | 12 | 2 | 45 | The 20-item Geriatric Depression Scale | NA |
| Vankova, et al. (2014) ^43^ | AE: 83.38±8.23 CON: 82.85±7.87 | AE: 79(76/3) CON: 83(73/10) | AE: 5.71±3.84 CON: 4.86±3.15 | >5 | AE: foxtrot, waltz, cha-cha, cancan, and so forth in a combination; CON: regular activities of the nursing home | 12 | 1 | 60 | The 15-item Geriatric Depression Scale | NA |
| Williams, et al. (2008) ^44^ | 87.9±5.95 | 45(40/5) | Mix: 12.18±5 Walk: 11.05±2.79 CON: 14.58±5.75 | >7 | Mix: strength, balance and flexibility exercises; Walking: Walking pace was individualized according to the participant’s ability; CON: casual conversation | 16 | 5 | Mix: 20 Walking: 30 | The Cornell Scale for Depression in Dementia | NA |
| Yang, et al. (2005) ^45^ | Qigong: 72.58 ± 5.41 CON: 72.67 ± 7.49 | Qigong: 19(13/6) CON: 21(19/2) | Qigong: 11.26 ± 6.07 CON: 10.1 ± 5.62 | NA | Qigong: Korean Qi-therapy (called Chun Soo energy healing); CON: routine activities | 4 | 2 | 20 | Profile of Mood States-depression-dejection | NA |
| Yeh, et al. (2010) ^46^ | Tai chi: 65 ± 6 CON: 66 ± 6 | Tai chi: 5(2/3) CON: 5(2/3) | Tai chi: 14 ± 8.75 CON: 12 ± 3.75 | >15 | Tai chi: emphasized gentle movement, relaxation, meditation, and breathing techniques; CON: usual care alone | 12 | 2 | 60 | Centre for Epidemiologic Studies for Depression scale | NA |
| Yu, et al. (2023) ^47^ | High Walking: 59.6±4.6 Moderate Walking: 60.6±3.1 CON: 60.5±7.3 | High Walking: 10 (6/4) Moderate Walking: 10 (8/2) CON: 10 (8/2) | High Walking: 27.9±7.6 Moderate Walking: 27.5±8.2 CON: 27.8±7.5 | >13 | High Walking: walking, 7 metabolic equivalents; Moderate Walking: walking, 3.5 metabolic equivalents; CON: no intervention | 12 | 3 | High: 25  Moderate: 50 | Beck Depression Inventory | NA |

*P* exercise period; *F* exercise frequency; *T* single session time; *NA* not available; *AE* Aerobic Exercise, *Mix* Mixed exercise, RT Resistance Training, *TC* Tai Chi.

# Supplementary 4: Risk of Bias

## Table 4.1 The risk of bias assessment for the individual included studies

| Study | 1 | 2 | 3 | 4 | 5 | 6 | 7 | 8 | 9 | 10 | 11 | Total |
| --- | --- | --- | --- | --- | --- | --- | --- | --- | --- | --- | --- | --- |
| Antunes, et al. (2005) ^1^ | Yes | Yes | No | Yes | No | No | No | Yes | Yes | Yes | Yes | 6 |
| Bernard, et al. (2015) ^2^ | Yes | Yes | No | Yes | No | No | No | Yes | Yes | Yes | Yes | 6 |
| Boström, et al. (2016) ^3^ | Yes | Yes | Yes | Yes | No | No | Yes | Yes | Yes | Yes | Yes | 8 |
| Bouaziz, et al. (2019) ^4^ | Yes | Yes | No | Yes | No | Yes | Yes | Yes | No | Yes | Yes | 7 |
| Brenes, et al. (2007) ^5^ | Yes | Yes | No | Yes | No | No | Yes | Yes | No | Yes | Yes | 6 |
| Brown, et al. (2009) ^6^ | Yes | Yes | No | Yes | No | No | No | No | No | Yes | Yes | 4 |
| Chen, et al. (2009) ^7^ | Yes | Yes | Yes | Yes | No | No | No | Yes | No | Yes | Yes | 6 |
| Cheng, et al. (2012) ^8^ | Yes | Yes | No | Yes | No | No | No | Yes | No | Yes | Yes | 5 |
| Chin A Paw, et al. (2004) ^9^ | Yes | Yes | No | Yes | No | No | Yes | No | Yes | Yes | Yes | 6 |
| Choi, et al. (2018) ^10^ | Yes | Yes | No | Yes | No | No | Yes | No | No | Yes | Yes | 5 |
| Chou, et al. (2004) ^11^ | Yes | Yes | No | Yes | No | Yes | No | Yes | No | Yes | Yes | 6 |
| Clegg, et al. (2014) ^12^ | Yes | Yes | Yes | Yes | No | No | Yes | No | Yes | Yes | No | 6 |
| de Lima, et al. (2019) ^13^ | Yes | Yes | No | Yes | No | No | Yes | Yes | Yes | Yes | Yes | 7 |
| Emery, et al. (1990) ^14^ | Yes | Yes | No | Yes | No | No | No | Yes | No | Yes | Yes | 4 |
| Eyre, et al. (2017) ^15^ | Yes | Yes | No | Yes | Yes | No | No | No | No | Yes | Yes | 5 |
| Fakhari (2017) ^16^ | Yes | Yes | No | Yes | No | No | No | Yes | No | Yes | Yes | 5 |
| Gary, et al. (2004) ^17^ | Yes | Yes | No | Yes | No | No | No | Yes | No | Yes | Yes | 5 |
| Gusi, et al. (2008) ^18^ | Yes | Yes | No | Yes | No | No | No | No | No | Yes | Yes | 4 |
| Hsu, et al. (2016) ^19^ | Yes | Yes | No | Yes | No | No | No | Yes | Yes | Yes | Yes | 6 |
| Huang, et al. (2015) ^20^ | Yes | Yes | No | Yes | No | No | Yes | Yes | Yes | Yes | Yes | 7 |
| Ibrahim, et al. (2023) ^21^ | Yes | Yes | No | Yes | Yes | No | Yes | Yes | No | Yes | Yes | 7 |
| Kim, et al. (2019) ^22^ | Yes | Yes | No | Yes | No | No | No | Yes | No | Yes | Yes | 5 |
| Kohut, et al. (2005) ^23^ | Yes | Yes | No | Yes | No | No | Yes | Yes | No | Yes | Yes | 6 |
| Lin, et al. (2007) ^24^ | Yes | Yes | No | Yes | No | No | Yes | No | No | Yes | Yes | 5 |
| Lincoln, et al. (2011) ^25^ | Yes | Yes | No | Yes | No | No | No | Yes | No | Yes | Yes | 5 |
| Lok, et al. (2017) ^26^ | Yes | Yes | No | Yes | No | No | Yes | Yes | Yes | Yes | Yes | 7 |
| McNeil, et al. (1991) ^27^ | Yes | Yes | No | Yes | No | No | No | Yes | Yes | Yes | Yes | 6 |
| Moraes, et al. (2020) ^28^ | Yes | Yes | No | Yes | No | No | Yes | No | No | Yes | Yes | 5 |
| Netz, et al. (1994) ^29^ | Yes | Yes | No | Yes | No | No | No | Yes | No | Yes | Yes | 5 |
| Ramanathan, et al. (2017) ^30^ | Yes | Yes | No | Yes | No | No | No | Yes | No | Yes | Yes | 5 |
| Roswiyani, et al. (2020) ^31^ | Yes | Yes | Yes | Yes | No | No | No | Yes | Yes | Yes | Yes | 7 |
| Sahin, et al. (2018) ^32^ | Yes | Yes | No | Yes | No | No | No | Yes | No | Yes | Yes | 5 |
| Shahidi, et al. (2011) ^33^ | Yes | Yes | No | Yes | No | No | No | Yes | No | Yes | Yes | 5 |
| Sims, et al. (2006) ^34^ | Yes | Yes | No | Yes | No | No | Yes | Yes | No | Yes | Yes | 6 |
| Sims, et al. (2009) ^35^ | Yes | Yes | No | Yes | No | No | No | Yes | No | Yes | Yes | 5 |
| Singh, et al. (1997) ^36^ | Yes | Yes | No | Yes | No | No | Yes | Yes | No | Yes | Yes | 6 |
| Singh, et al. (2005) ^37^ | Yes | Yes | Yes | Yes | No | No | Yes | Yes | No | Yes | Yes | 7 |
| Song, et al. (2019) ^38^ | Yes | Yes | No | Yes | No | No | Yes | No | No | Yes | Yes | 5 |
| Tsang, et al. (2003) ^39^ | Yes | Yes | No | Yes | No | No | No | Yes | No | Yes | Yes | 4 |
| Tsang, et al. (2006) ^40^ | Yes | Yes | No | Yes | Yes | No | Yes | Yes | No | Yes | Yes | 7 |
| Tsang, et al. (2013) ^41^ | Yes | Yes | No | Yes | No | No | Yes | Yes | Yes | Yes | Yes | 7 |
| Vahlberg, et al. (2017) ^42^ | Yes | Yes | Yes | Yes | No | Yes | No | No | No | Yes | Yes | 6 |
| Vankova, et al. (2014) ^43^ | Yes | Yes | Yes | Yes | No | No | Yes | Yes | No | Yes | Yes | 7 |
| Williams, et al. (2008) ^44^ | Yes | Yes | No | Yes | No | No | Yes | Yes | Yes | Yes | Yes | 7 |
| Yang, et al. (2005) ^45^ | Yes | Yes | No | Yes | No | No | No | Yes | No | Yes | Yes | 5 |
| Yeh, et al. (2010) ^46^ | Yes | Yes | No | Yes | No | No | Yes | Yes | No | Yes | Yes | 6 |
| Yu, et al. (2023) ^47^ | Yes | Yes | No | Yes | No | No | Yes | No | No | Yes | Yes | 5 |

1: eligibility criteria were specified; 2: subjects were randomly allocated to groups (in a crossover study, subjects were randomly allocated an order in which treatments were received); 3: allocation was concealed; 4: the groups were similar at baseline regarding the most important prognostic indicators; 5: there was blinding of all subjects; 6: there was blinding of all therapists who administered the therapy; 7: there was blinding of all assessors who measured at least one key outcome; 8: measures of at least one key outcome were obtained from more than 85% of the subjects initially allocated to groups; 9: all subjects for whom outcome measures were available received the treatment or control condition as allocated or, where this was not the case, data for at least one key outcome was analysed by “intention to treat”; 10: the results of between-group statistical comparisons are reported for at least one key outcome; 11: the study provides both point measures and measures of variability for at least one key outcome.

**List of included studies**

1. Antunes HKM, Stella SG, Santos RF, Bueno OFA, de Mello MT. Depression, anxiety and quality of life scores in seniors after an endurance exercise program. Braz J Psychiatry. 2005;27(4):266-271. doi. https://pubmed.ncbi.nlm.nih.gov/16358106.

2. Bernard P, Ninot G, Bernard PL, et al. Effects of a six-month walking intervention on depression in inactive post-menopausal women: a randomized controlled trial. Aging Ment Health. 2015;19(6):485-492. doi: 10.1080/13607863.2014.948806.

3. Boström G, Conradsson M, Hörnsten C, et al. Effects of a high-intensity functional exercise program on depressive symptoms among people with dementia in residential care: a randomized controlled trial. Int J Geriatr Psychiatry. 2016;31(8):868-878. doi: 10.1002/gps.4401.

4. Bouaziz W, Schmitt E, Vogel T, et al. Effects of a short-term Interval Aerobic Training Programme with active Recovery bouts (IATP-R) on cognitive and mental health, functional performance and quality of life: A randomised controlled trial in sedentary seniors. Int J Clin Pract. 2019;73(1):e13219. doi: 10.1111/ijcp.13219.

5. Brenes GA, Williamson JD, Messier SP, et al. Treatment of minor depression in older adults: a pilot study comparing sertraline and exercise. Aging Ment Health. 2007;11(1):61-68. doi. https://pubmed.ncbi.nlm.nih.gov/17164159.

6. Brown AK, Liu-Ambrose T, Tate R, Lord SR. The effect of group-based exercise on cognitive performance and mood in seniors residing in intermediate care and self-care retirement facilities: a randomised controlled trial. Br J Sports Med. 2009;43(8):608-614. doi: 10.1136/bjsm.2008.049882.

7. Chen K-M, Chen M-H, Chao H-C, Hung H-M, Lin H-S, Li C-H. Sleep quality, depression state, and health status of older adults after silver yoga exercises: cluster randomized trial. Int J Nurs Stud. 2009;46(2):154-163. doi: 10.1016/j.ijnurstu.2008.09.005.

8. Cheng S-T, Chow PK, Yu ECS, Chan ACM. Leisure activities alleviate depressive symptoms in nursing home residents with very mild or mild dementia. Am J Geriatr Psychiatry. 2012;20(10):904-908. doi: 10.1097/JGP.0b013e3182423988.

9. Chin A Paw MJM, van Poppel MNM, Twisk JWR, van Mechelen W. Effects of resistance and all-round, functional training on quality of life, vitality and depression of older adults living in long-term care facilities: a 'randomized' controlled trial [ISRCTN87177281]. BMC Geriatr. 2004;4:5. doi. https://pubmed.ncbi.nlm.nih.gov/15233841.

10. Choi M-J, Sohng K-Y. The effects of floor-seated exercise program on physical fitness, depression, and sleep in older adults: A cluster randomized controlled trial. International journal of gerontology. 2018;12(2):116-121. doi.

11. Chou K-L, Lee PWH, Yu ECS, et al. Effect of Tai Chi on depressive symptoms amongst Chinese older patients with depressive disorders: a randomized clinical trial. Int J Geriatr Psychiatry. 2004;19(11):1105-1107. doi. https://pubmed.ncbi.nlm.nih.gov/15497192.

12. Clegg A, Barber S, Young J, Iliffe S, Forster A. The Home-based Older People's Exercise (HOPE) trial: a pilot randomised controlled trial of a home-based exercise intervention for older people with frailty. Age Ageing. 2014;43(5):687-695. doi: 10.1093/ageing/afu033.

13. de Lima TA, Ferreira-Moraes R, Alves WMGdC, et al. Resistance training reduces depressive symptoms in elderly people with Parkinson disease: A controlled randomized study. Scand J Med Sci Sports. 2019;29(12):1957-1967. doi: 10.1111/sms.13528.

14. Emery CF, Gatz M. Psychological and cognitive effects of an exercise program for community-residing older adults. Gerontologist. 1990;30(2):184-188. doi. https://pubmed.ncbi.nlm.nih.gov/2347498.

15. Eyre HA, Siddarth P, Acevedo B, et al. A randomized controlled trial of Kundalini yoga in mild cognitive impairment. Int Psychogeriatr. 2017;29(4):557-567. doi: 10.1017/S1041610216002155.

16. Fakhari M. Effects of Tai Chi exercise on depression in older adults: A randomized controlled trial. Bali Medical Journal. 2017;6(3):679-683. doi.

17. Gary RA, Sueta CA, Dougherty M, et al. Home-based exercise improves functional performance and quality of life in women with diastolic heart failure. Heart Lung. 2004;33(4):210-218. doi. https://pubmed.ncbi.nlm.nih.gov/15252410.

18. Gusi N, Reyes MC, Gonzalez-Guerrero JL, Herrera E, Garcia JM. Cost-utility of a walking programme for moderately depressed, obese, or overweight elderly women in primary care: a randomised controlled trial. BMC Public Health. 2008;8:231. doi: 10.1186/1471-2458-8-231.

19. Hsu C-Y, Moyle W, Cooke M, Jones C. Seated Tai Chi versus usual activities in older people using wheelchairs: A randomized controlled trial. Complement Ther Med. 2016;24:1-6. doi: 10.1016/j.ctim.2015.11.006.

20. Huang T-T, Liu C-B, Tsai Y-H, Chin Y-F, Wong C-H. Physical fitness exercise versus cognitive behavior therapy on reducing the depressive symptoms among community-dwelling elderly adults: A randomized controlled trial. Int J Nurs Stud. 2015;52(10):1542-1552. doi: 10.1016/j.ijnurstu.2015.05.013.

21. Ibrahim AA, Hussein HM, Ali MS, et al. A randomized controlled trial examining the impact of low vs. moderate-intensity aerobic training in post-discharge COVID-19 older subjects. Eur Rev Med Pharmacol Sci. 2023;27(9):4280-4291. doi: 10.26355/eurrev_202305_32338.

22. Kim Y-S, O'Sullivan DM, Shin S-K. Can 24 weeks strength training reduce feelings of depression and increase neurotransmitter in elderly females? Exp Gerontol. 2019;115:62-68. doi: 10.1016/j.exger.2018.11.009.

23. Kohut ML, Lee W, Martin A, et al. The exercise-induced enhancement of influenza immunity is mediated in part by improvements in psychosocial factors in older adults. Brain Behav Immun. 2005;19(4):357-366. doi. https://pubmed.ncbi.nlm.nih.gov/15944076.

24. Lin M-R, Wolf SL, Hwang H-F, Gong S-Y, Chen C-Y. A randomized, controlled trial of fall prevention programs and quality of life in older fallers. J Am Geriatr Soc. 2007;55(4):499-506. doi. https://pubmed.ncbi.nlm.nih.gov/17397426.

25. Lincoln AK, Shepherd A, Johnson PL, Castaneda-Sceppa C. The impact of resistance exercise training on the mental health of older Puerto Rican adults with type 2 diabetes. J Gerontol B Psychol Sci Soc Sci. 2011;66(5):567-570. doi: 10.1093/geronb/gbr034.

26. Lok N, Lok S, Canbaz M. The effect of physical activity on depressive symptoms and quality of life among elderly nursing home residents: Randomized controlled trial. Arch Gerontol Geriatr. 2017;70:92-98. doi: 10.1016/j.archger.2017.01.008.

27. McNeil JK, LeBlanc EM, Joyner M. The effect of exercise on depressive symptoms in the moderately depressed elderly. Psychol Aging. 1991;6(3):487-488. doi. https://pubmed.ncbi.nlm.nih.gov/1930766.

28. Moraes HS, Silveira HS, Oliveira NA, et al. Is Strength Training as Effective as Aerobic Training for Depression in Older Adults? A Randomized Controlled Trial. Neuropsychobiology. 2020;79(2):141-149. doi: 10.1159/000503750.

29. Netz Y, Yaretzki A, Salganik I, Jacob T, Finkeltov B, Argov E. The effect of supervised physical activity on cognitive and affective state of geriatric and psychogeriatric in-patients. Clinical gerontologist. 1994;15(1):47-56. doi.

30. Ramanathan M, Bhavanani AB, Trakroo M. Effect of a 12-week yoga therapy program on mental health status in elderly women inmates of a hospice. Int J Yoga. 2017;10(1):24-28. doi: 10.4103/0973-6131.186156.

31. Roswiyani R, Hiew CH, Witteman CLM, Satiadarma MP, Spijker J. Art activities and qigong exercise for the well-being of older adults in nursing homes in Indonesia: a randomized controlled trial. Aging Ment Health. 2020;24(10):1569-1578. doi: 10.1080/13607863.2019.1617239.

32. Sahin UK, Kirdi N, Bozoglu E, et al. Effect of low-intensity versus high-intensity resistance training on the functioning of the institutionalized frail elderly. Int J Rehabil Res. 2018;41(3):211-217. doi: 10.1097/MRR.0000000000000285.

33. Shahidi M, Mojtahed A, Modabbernia A, et al. Laughter yoga versus group exercise program in elderly depressed women: a randomized controlled trial. Int J Geriatr Psychiatry. 2011;26(3):322-327. doi: 10.1002/gps.2545.

34. Sims J, Hill K, Davidson S, Gunn J, Huang N. Exploring the feasibility of a community-based strength training program for older people with depressive symptoms and its impact on depressive symptoms. BMC Geriatr. 2006;6:18. doi. https://pubmed.ncbi.nlm.nih.gov/17134517.

35. Sims J, Galea M, Taylor N, et al. Regenerate: assessing the feasibility of a strength-training program to enhance the physical and mental health of chronic post stroke patients with depression. Int J Geriatr Psychiatry. 2009;24(1):76-83. doi: 10.1002/gps.2082.

36. Singh NA, Clements KM, Fiatarone MA. A randomized controlled trial of progressive resistance training in depressed elders. J Gerontol A Biol Sci Med Sci. 1997;52(1):M27-M35. doi. https://pubmed.ncbi.nlm.nih.gov/9008666.

37. Singh NA, Stavrinos TM, Scarbek Y, Galambos G, Liber C, Fiatarone Singh MA. A randomized controlled trial of high versus low intensity weight training versus general practitioner care for clinical depression in older adults. J Gerontol A Biol Sci Med Sci. 2005;60(6):768-776. doi. https://pubmed.ncbi.nlm.nih.gov/15983181.

38. Song D, Yu DSF. Effects of a moderate-intensity aerobic exercise programme on the cognitive function and quality of life of community-dwelling elderly people with mild cognitive impairment: A randomised controlled trial. Int J Nurs Stud. 2019;93. doi: 10.1016/j.ijnurstu.2019.02.019.

39. Tsang HWH, Mok CK, Au Yeung YT, Chan SYC. The effect of Qigong on general and psychosocial health of elderly with chronic physical illnesses: a randomized clinical trial. Int J Geriatr Psychiatry. 2003;18(5):441-449. doi. https://pubmed.ncbi.nlm.nih.gov/12766922.

40. Tsang HWH, Fung KMT, Chan ASM, Lee G, Chan F. Effect of a qigong exercise programme on elderly with depression. Int J Geriatr Psychiatry. 2006;21(9):890-897. doi. https://pubmed.ncbi.nlm.nih.gov/16955451.

41. Tsang HWH, Tsang WWN, Jones AYM, et al. Psycho-physical and neurophysiological effects of qigong on depressed elders with chronic illness. Aging Ment Health. 2013;17(3):336-348. doi: 10.1080/13607863.2012.732035.

42. Vahlberg B, Cederholm T, Lindmark B, Zetterberg L, Hellström K. Short-term and long-term effects of a progressive resistance and balance exercise program in individuals with chronic stroke: a randomized controlled trial. Disabil Rehabil. 2017;39(16):1615-1622. doi: 10.1080/09638288.2016.1206631.

43. Vankova H, Holmerova I, Machacova K, Volicer L, Veleta P, Celko AM. The effect of dance on depressive symptoms in nursing home residents. J Am Med Dir Assoc. 2014;15(8):582-587. doi: 10.1016/j.jamda.2014.04.013.

44. Williams CL, Tappen RM. Exercise training for depressed older adults with Alzheimer's disease. Aging Ment Health. 2008;12(1):72-80. doi: 10.1080/13607860701529932.

45. Yang KH, Kim YH, Lee MS. Efficacy of Qi-therapy (external Qigong) for elderly people with chronic pain. Int J Neurosci. 2005;115(7):949-963. doi. https://pubmed.ncbi.nlm.nih.gov/16051542.

46. Yeh GY, Roberts DH, Wayne PM, Davis RB, Quilty MT, Phillips RS. Tai chi exercise for patients with chronic obstructive pulmonary disease: a pilot study. Respir Care. 2010;55(11):1475-1482. doi. https://pubmed.ncbi.nlm.nih.gov/20979675.

47. Yu DJ, Yu AP, Leung CK, et al. Comparison of moderate and vigorous walking exercise on reducing depression in middle-aged and older adults: A pilot randomized controlled trial. Eur J Sport Sci. 2023;23(6):1018-1027. doi: 10.1080/17461391.2022.2079424.

# Supplementary 5: Evaluation of inconsistency

**Table 5.1 Details of SIDE splitting results**

| Comparison | k | prop | NMA | |  | direct | |  | indir | |  | diff | | p |
| --- | --- | --- | --- | --- | --- | --- | --- | --- | --- | --- | --- | --- | --- | --- |
|  |  |  | TE | seTE |  | TE | seTE |  | TE | seTE |  | TE | seTE |  |
| AE vs CON | 9 | 0.86911402 | -0.6712145 | 0.16111489 |  | -0.7308872 | 0.17282132 |  | -0.2749736 | 0.44533735 |  | -0.4559136 | 0.47769505 | 0.33987967 |
| AE vs Mix | 2 | 0.27859188 | -0.3763819 | 0.20664022 |  | -0.1487747 | 0.39149897 |  | -0.4642788 | 0.24329004 |  | 0.31550408 | 0.46093545 | 0.49366803 |
| AE vs Qigong | 0 | 0 | 0.00820612 | 0.24431069 |  | NA | NA |  | 0.00820612 | 0.24431069 |  | NA | NA | NA |
| AE vs RT | 1 | 0.10874651 | 0.07015512 | 0.21711141 |  | 0.91693904 | 0.65837751 |  | -0.0331654 | 0.22997574 |  | 0.95010447 | 0.69738783 | 0.17307924 |
| AE vs TC | 0 | 0 | -0.008099 | 0.29050704 |  | NA | NA |  | -0.008099 | 0.29050704 |  | NA | NA | NA |
| AE vs Walking | 0 | 0 | 0.20258665 | 0.24421361 |  | NA | NA |  | 0.20258665 | 0.24421361 |  | NA | NA | NA |
| AE vs Yoga | 1 | 0.21162667 | 0.03357454 | 0.24830021 |  | 0.30479409 | 0.53974906 |  | -0.0392302 | 0.27964762 |  | 0.34402425 | 0.6078913 | 0.571441 |
| Mix vs CON | 11 | 0.8714937 | -0.2948326 | 0.14799901 |  | -0.2064869 | 0.15853556 |  | -0.8939683 | 0.4128541 |  | 0.68748141 | 0.44224658 | 0.12006029 |
| Qigong vs CON | 8 | 1 | -0.6794207 | 0.18365649 |  | -0.6794207 | 0.18365649 |  | NA | NA |  | NA | NA | NA |
| RT vs CON | 12 | 0.90878116 | -0.7413697 | 0.15379438 |  | -0.6785456 | 0.16132839 |  | -1.3672633 | 0.50921147 |  | 0.68871769 | 0.53415651 | 0.19727449 |
| TC vs CON | 6 | 1 | -0.6631155 | 0.24173608 |  | -0.6631155 | 0.24173608 |  | NA | NA |  | NA | NA | NA |
| Walking vs CON | 9 | 0.96286503 | -0.8738012 | 0.18459002 |  | -0.8843389 | 0.1881159 |  | -0.6005704 | 0.95789225 |  | -0.2837685 | 0.9761891 | 0.77128832 |
| Yoga vs CON | 6 | 0.95801252 | -0.7047891 | 0.20137346 |  | -0.6932455 | 0.20573901 |  | -0.9681749 | 0.98274835 |  | 0.27492938 | 1.00405322 | 0.78422331 |
| Mix vs Qigong | 0 | 0 | 0.38458804 | 0.23586736 |  | NA | NA |  | 0.38458804 | 0.23586736 |  | NA | NA | NA |
| Mix vs RT | 2 | 0.25784794 | 0.44653703 | 0.20283975 |  | 0.46429432 | 0.3994581 |  | 0.44036757 | 0.2354543 |  | 0.02392676 | 0.46368686 | 0.95884654 |
| Mix vs TC | 0 | 0 | 0.3682829 | 0.28344319 |  | NA | NA |  | 0.3682829 | 0.28344319 |  | NA | NA | NA |
| Mix vs Walking | 1 | 0.16886819 | 0.57896857 | 0.22947111 |  | 0.02952037 | 0.55841114 |  | 0.69060468 | 0.2517057 |  | -0.6610843 | 0.61251838 | 0.28045894 |
| Mix vs Yoga | 0 | 0 | 0.40995646 | 0.24894209 |  | NA | NA |  | 0.40995646 | 0.24894209 |  | NA | NA | NA |
| Qigong vs RT | 0 | 0 | 0.06194899 | 0.23954627 |  | NA | NA |  | 0.06194899 | 0.23954627 |  | NA | NA | NA |
| Qigong vs TC | 0 | 0 | -0.0163051 | 0.3035886 |  | NA | NA |  | -0.0163051 | 0.3035886 |  | NA | NA | NA |
| Qigong vs Walking | 0 | 0 | 0.19438053 | 0.26039044 |  | NA | NA |  | 0.19438053 | 0.26039044 |  | NA | NA | NA |
| Qigong vs Yoga | 0 | 0 | 0.02536842 | 0.27254536 |  | NA | NA |  | 0.02536842 | 0.27254536 |  | NA | NA | NA |
| RT vs TC | 0 | 0 | -0.0782541 | 0.28651185 |  | NA | NA |  | -0.0782541 | 0.28651185 |  | NA | NA | NA |
| RT vs Walking | 0 | 0 | 0.13243153 | 0.23956578 |  | NA | NA |  | 0.13243153 | 0.23956578 |  | NA | NA | NA |
| RT vs Yoga | 0 | 0 | -0.0365806 | 0.25292809 |  | NA | NA |  | -0.0365806 | 0.25292809 |  | NA | NA | NA |
| TC vs Walking | 0 | 0 | 0.21068566 | 0.30415425 |  | NA | NA |  | 0.21068566 | 0.30415425 |  | NA | NA | NA |
| TC vs Yoga | 0 | 0 | 0.04167356 | 0.31462295 |  | NA | NA |  | 0.04167356 | 0.31462295 |  | NA | NA | NA |
| Walking vs Yoga | 0 | 0 | -0.1690121 | 0.27310832 |  | NA | NA |  | -0.1690121 | 0.27310832 |  | NA | NA | NA |

*NA* not available, *k* Number of studies providing direct evidence, *prop* Direct evidence proportion, *nma* Estimated treatment effect (SMD) in network meta-analysis, *direct* Estimated treatment effect (SMD) derived from direct evidence, *indir.* Estimated treatment effect (SMD) derived from indirect evidence, *Diff* Difference between direct and indirect treatment estimates, *p* p-value of test for disagreement (direct versus indirect).

.

# Supplementary 6: Publication bias

As shown in the figure below, the funnel plot had good symmetry, and the linear fitting line (green) is not perpendicular to the 0 quadrant, and the result of Egger test showed the p=0.059. Therefore, no small study effect was found for the primary outcome.

Figure 6.1 The funnel plot of change of depression symptoms. *AE* Aerobic Exercise, *Mix* Mixed exercise, RT Resistance Training, *TC* Tai Chi.

Supplementary 7: Grading the evidence for depression symptoms of the network meta-analysis using CINeMA

## 7.1 Summary of study limitations of the included studies


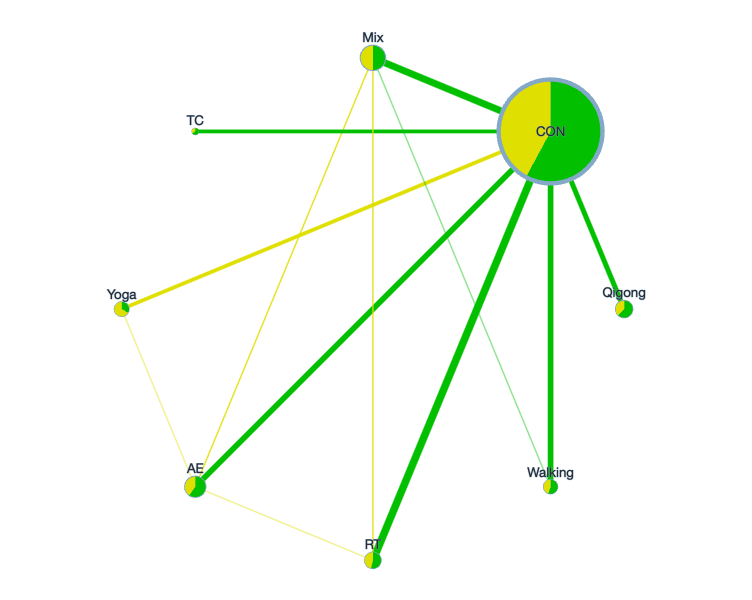


Figure 7.1 Network plot of study limitations of the included studies. Node size by equal size, node color by RoB. The colors in the circles indicate the percentage of low RoB studies (green), moderate RoB studies (yellow), high RoB studies (red) about each physical activity type. Edge width by sample size. Edge color by average RoB. The colors of the lines indicate the summative RoB assessment of each comparison. Low RoB is green, moderate RoB is yellow, high RoB is red. *AE* Aerobic Exercise, *CON* Control group, *Mix* Mixed exercise, RT Resistance Training, *TC* Tai Chi.

## 7.2 Reasons for downgrading

Based on the recommendations of the CINeMA online document (https://cinema.ispm.unibe.ch/), we only graded the results of the depression symptoms, and judged whether each module needs to be downgraded according to the following criteria.

***With-study bias***

We classified the quality evaluation results (Supplementary 4) of each included study into low-risk (PEDro scale ≥6 points), moderate-risk (PDEro scale 4-6 points), and high-risk (PDEro scale <4 points). We selected the rule is average RoB. No need to downgrade when the result was “no concerns”, downgrade one level when “some concerns” and downgrade two level “major concerns”.

***Across-study bias (publication bias)***

Our search was relatively comprehensive, including published and unpublished studies. Due to the language is not limited, Chinese studies were also included in the scope of our inclusion. Even if it is possible that we missed other small unpublished experiments, then it does not seem to affect our results. In Supplementary 6, we evaluated the outcome of publication bias, and comparison-adjusted funnel plots for exercise types showed no evidence of asymmetry. Therefore, the outcome was deemed to have no publication bias.

***Indirectness***

In Supplementary 5, we performed a point inconsistency test on the outcome measures by the SIDE test and found no significant differences. Therefore, no indirectness was assumed and no comparison was downgraded for this reason.

***Imprecision***

The outcome (depression symptoms) of this network meta-analysis is a continuous variable, and the effect size measure for continuous outcomes chooses the standardized mean difference (SMD) of the change score (end-point minus baseline score) because the studies use different rating scales or units (Supplementary 6). Therefore, for CON comparisons the clinically meaningful threshold was set at a standardized mean difference of higher or lower than 0, and for the comparisons of the two types of exercise, the threshold was set at SMD -0.1 and 0.1. If the confidence interval crossed one threshold, it will be downgraded by one level, and two thresholds will be downgraded by two levels.

***Heterogeneity***

For heterogeneity, we used the same threshold as the above clinically significant threshold and follow the recommendations automatically provided by CINeMA (https://cinema.ispm.unibe.ch/). No need to downgrade when the result was “no concerns”, downgrade one level when “some concerns” and downgrade two level when “major concerns”.

***Incoherence***

For incoherence, we will use global and local methods to test the inconsistency of the research results. For global inconsistency, we evaluated inconsistency statistically using the design-by-treatment test. In addition, we will assessment of local inconsistency by separating indirect from direct evidence (SIDE test) using the R netmeta package (Supplementary 12). No need to downgrade when p >0.1, downgrade one level when p was 0.05-0.1 and downgrade two level when p <0.05 .

***Summarising judgments across the 6 domains***

Τhe final output of CINeMA is a table with the level of concern for each of the 6 domains. we choose to summarise judgments across domains using the 4 levels of confidence of the GRADE approach: very low, low, moderate, or high.(Puhan et al., 2014) Due to factors that may reduce the confidence in a treatment effect may affect more than 1 domain. Indirectness includes consideration of intransitivity, which is manifested as statistical incoherence in the data. Heterogeneity will increase the imprecision of treatment effect, and may be related to the variability of bias within the study or the existence of reporting bias. In addition, in the presence of heterogeneity, the ability to detect important discontinuities will be reduced.(Veroniki, Mavridis, Higgins, & Salanti, 2014) Therefore, the 6 CINeMA domains should be considered jointly rather than in isolation to avoid downgrading the overall level of confidence more than once for related concerns.

## 7.3 CINeMA for the primary outcome “depression symptoms”

| **Comparison** | **Number of studies** | **Within-study bias** | **Reporting bias** | **Indirectness** | **Imprecision** | **Heterogeneity** | **Incoherence** | **Confidence rating** | **Reason(s) for downgrading** |
| --- | --- | --- | --- | --- | --- | --- | --- | --- | --- |
| AE:CON | 9 | No concerns | Low risk | No concerns | No concerns | No concerns | No concerns | High | [] |
| AE:Mix | 2 | Some concerns | Low risk | No concerns | No concerns | Major concerns | No concerns | Very low | ["Within-study bias","Heterogeneity"] |
| AE:RT | 1 | No concerns | Low risk | No concerns | No concerns | No concerns | No concerns | High | [] |
| AE:Yoga | 1 | Some concerns | Low risk | No concerns | Major concerns | No concerns | No concerns | Very low | ["Within-study bias","Imprecision"] |
| CON:Mix | 11 | No concerns | Low risk | No concerns | Some concerns | No concerns | No concerns | Moderate | ["Imprecision"] |
| CON:Qigong | 8 | No concerns | Low risk | No concerns | No concerns | No concerns | No concerns | High | [] |
| CON:RT | 12 | No concerns | Low risk | No concerns | No concerns | No concerns | No concerns | High | [] |
| CON:TC | 6 | No concerns | Low risk | No concerns | No concerns | Major concerns | No concerns | Low | ["Heterogeneity"] |
| CON:Walking | 9 | No concerns | Low risk | No concerns | No concerns | No concerns | No concerns | High | [] |
| CON:Yoga | 6 | Some concerns | Low risk | No concerns | No concerns | No concerns | No concerns | Moderate | ["Within-study bias"] |
| Mix:RT | 2 | No concerns | Low risk | No concerns | No concerns | Some concerns | No concerns | Moderate | ["Heterogeneity"] |
| Mix:Walking | 1 | No concerns | Low risk | No concerns | No concerns | Major concerns | No concerns | Low | ["Heterogeneity"] |
| AE:Qigong | 0 | No concerns | Low risk | No concerns | No concerns | No concerns | No concerns | High | [] |
| AE:TC | 0 | No concerns | Low risk | No concerns | No concerns | No concerns | No concerns | High | [] |
| AE:Walking | 0 | No concerns | Low risk | No concerns | No concerns | No concerns | No concerns | High | [] |
| Mix:Qigong | 0 | No concerns | Low risk | No concerns | No concerns | No concerns | No concerns | High | [] |
| Mix:TC | 0 | No concerns | Low risk | No concerns | Major concerns | No concerns | No concerns | Low | ["Imprecision"] |
| Mix:Yoga | 0 | Some concerns | Low risk | No concerns | Some concerns | No concerns | No concerns | Low | ["Within-study bias","Imprecision"] |
| Qigong:RT | 0 | No concerns | Low risk | No concerns | No concerns | No concerns | No concerns | High | [] |
| Qigong:TC | 0 | No concerns | Low risk | No concerns | No concerns | No concerns | No concerns | High | [] |
| Qigong:Walking | 0 | No concerns | Low risk | No concerns | No concerns | No concerns | No concerns | High | [] |
| Qigong:Yoga | 0 | No concerns | Low risk | No concerns | Some concerns | No concerns | No concerns | Moderate | ["Imprecision"] |
| RT:TC | 0 | No concerns | Low risk | No concerns | Some concerns | No concerns | No concerns | Moderate | ["Imprecision"] |
| RT:Walking | 0 | No concerns | Low risk | No concerns | No concerns | No concerns | No concerns | High | [] |
| RT:Yoga | 0 | Some concerns | Low risk | No concerns | No concerns | No concerns | No concerns | Moderate | ["Within-study bias"] |
| TC:Walking | 0 | No concerns | Low risk | No concerns | Major concerns | No concerns | No concerns | Low | ["Imprecision"] |
| TC:Yoga | 0 | Some concerns | Low risk | No concerns | Major concerns | No concerns | No concerns | Very low | ["Within-study bias","Imprecision"] |
| Walking:Yoga | 0 | Some concerns | Low risk | No concerns | No concerns | No concerns | No concerns | Moderate | ["Within-study bias"] |

*AE* Aerobic Exercise, *AQE* Aquatic Exercise, *BGT* Balance and Gait Training, *BGT_ECA* Balance and Gait Training with External Cue or Attention, *BGT_ICA* Balance and Gait Training with Internal Cue or Attention, *BWS_TT* Body Weight Support Treadmill Training, *CON* Control group, *CPP* Classic Physiotherapy Program, *DT_BGT* Dual Task Balance and Gait Training, *Mul_C* Multicomponent Exercise Program, *Mul_D* Multidisciplinary Exercise Program, *NW* Nordic Walking, *PT* Power Training, *RA_GT* Robotic Assisted Gait Training, *RT* Resistance Training, *TC* Tai Chi, *TT* Treadmill Training, *VR* Virtual Reality, *WBV* Whole Body Vibration.

**Supplementary 8:** Assessment of Connectivity, Consistency and Transitivity in Network Meta Dose-Response Analysis

***Connectivity***

Connectivity is a key assumption in network meta-dose analysis, and evidence of unconnectedness may lead to low statistical power and misleading results (Ter Veer, van Oijen, & van Laarhoven, 2019). Our results show that there is no phenomenon of poor connectivity (Figures 8.1, 2).

**Figure 8.1**. Treatment-level network. The first value indicates the specific intervention and the second one is the corresponding dose of that intervention. *AE* Aerobic Exercise, *Mix* Mixed exercise, RT Resistance Training, *TC* Tai Chi.

**Figure 8.2**. Agent-level network. *AE* Aerobic Exercise, *Mix* Mixed exercise, RT Resistance Training, *TC* Tai Chi.

**Consistency**

We analyzed the data with the consistency model and the unrelated mean effect model, and compared the differences in the deviation, the number of estimated parameters in the network, and the Deviance Informative Criterion (DIC) indicators of the two models. If these are similar, it means that our research has good consistency (Wheeler, Hickson, & Waller, 2010). Comparison of these parameters indicated good consistency across models (Table 8.1).

**Table 8.1.** Consistent and UME models fit comparison

| **Model** | **pD** | **Residual deviance** | **Deviance** | **DIC** | **SD** |
| --- | --- | --- | --- | --- | --- |
| Consistent | 101.9 | 124.424 | 303.007 | 404.4 | 0.339 |
| UME | 107.6 | 120.293 | 298.876 | 405.5 | 0.571 |

pD: Number of estimated parameters; DIC: Deviance Informative Criterion; SD: Standard Deviation; UME: Unrelated Mean Effects. Scientific literature indicated that the main indicator to assess the model fit is the DIC. As lower DIC, better fit.

**Transitivity**

We assessed transitivity via MBNMA node-splitting approach. This method splits and compares contributions for a particular treatment contrast into direct and indirect evidence (van Valkenhoef, Dias, Ades, & Welton, 2016). Similar effects denote good transitivity. Figures 8.3 and Table 8.2 below present the results for transitivity in this meta-analysis.

Table 8.2 Node-splitting analysis of inconsistency

| **Comparison** | **p-value** | **Median** | **2.50%** | **97.50%** |
| --- | --- | --- | --- | --- |
| RT_500 vs AE_500 | 0.553 |  |  |  |
| -> direct |  | 0.221 | -1.112 | 1.602 |
| -> indirect |  | 0.079 | -0.472 | 0.589 |
| -> MBNMA | | 0.088 | -0.414 | 0.569 |
|  |  |  |  |  |
| Mix_250 vs AE_500 | 0.564 |  |  |  |
| -> direct |  | 0.723 | -0.609 | 2.102 |
| -> indirect |  | 0.506 | -0.033 | 1.02 |
| -> MBNMA | | 0.566 | 0.046 | 1.024 |
|  |  |  |  |  |
| Yoga_750 vs Placebo_0 | 0.381 |  |  |  |
| -> direct |  | -1.307 | -2.124 | -0.533 |
| -> indirect |  | -0.679 | -1.324 | -0.044 |
| -> MBNMA | | -0.977 | -1.505 | -0.415 |
|  |  |  |  |  |
| Yoga_500 vs Placebo_0 | 0.959 |  |  |  |
| -> direct |  | -0.844 | -1.531 | -0.17 |
| -> indirect |  | -0.866 | -1.492 | -0.246 |
| -> MBNMA | | -0.88 | -1.315 | -0.405 |
|  |  |  |  |  |
| Yoga_250 vs Placebo_0 | 0.392 |  |  |  |
| -> direct |  | -0.238 | -1.458 | 0.888 |
| -> indirect |  | -0.895 | -1.402 | -0.384 |
| -> MBNMA | | -0.726 | -1.242 | -0.299 |
|  |  |  |  |  |
| Walking_750 vs Placebo_0 | 0.263 |  |  |  |
| -> direct |  | -1.536 | -2.374 | -0.728 |
| -> indirect |  | -0.748 | -1.316 | -0.255 |
| -> MBNMA | | -0.986 | -1.558 | -0.439 |
|  |  |  |  |  |
| Walking_500 vs Placebo_0 | 0.627 |  |  |  |
| -> direct |  | -0.989 | -1.556 | -0.428 |
| -> indirect |  | -0.685 | -1.346 | -0.011 |
| -> MBNMA | | -0.88 | -1.293 | -0.428 |
|  |  |  |  |  |
| Walking_250 vs Placebo_0 | 0.274 |  |  |  |
| -> direct |  | -0.206 | -1.007 | 0.595 |
| -> indirect |  | -0.941 | -1.437 | -0.443 |
| -> MBNMA | | -0.685 | -1.133 | -0.297 |
|  |  |  |  |  |
| TC_500 vs Placebo_0 | 0.401 |  |  |  |
| -> direct |  | -0.911 | -1.529 | -0.332 |
| -> indirect |  | -0.249 | -1.341 | 0.746 |
| -> MBNMA | | -0.755 | -1.283 | -0.175 |
|  |  |  |  |  |
| TC_250 vs Placebo_0 | 0.527 |  |  |  |
| -> direct |  | -0.29 | -1.475 | 0.874 |
| -> indirect |  | -0.756 | -1.295 | -0.204 |
| -> MBNMA | | -0.625 | -1.165 | -0.126 |
|  |  |  |  |  |
| RT_1200 vs Placebo_0 | 0.265 |  |  |  |
| -> direct |  | -0.066 | -1.304 | 1.222 |
| -> indirect |  | -1.258 | -1.848 | -0.491 |
| -> MBNMA | | -1.054 | -1.656 | -0.431 |
|  |  |  |  |  |
| RT_1000 vs Placebo_0 | 0.699 |  |  |  |
| -> direct |  | -0.865 | -2.012 | 0.216 |
| -> indirect |  | -1 | -1.545 | -0.42 |
| -> MBNMA | | -0.987 | -1.49 | -0.431 |
|  |  |  |  |  |
| RT_750 vs Placebo_0 | 0.014 |  |  |  |
| -> direct |  | -1.823 | -2.435 | -1.186 |
| -> indirect |  | -0.403 | -0.842 | 0 |
| -> MBNMA | | -0.882 | -1.289 | -0.426 |
|  |  |  |  |  |
| RT_250 vs Placebo_0 | 0.048 |  |  |  |
| -> direct |  | 0.042 | -0.507 | 0.573 |
| -> indirect |  | -1.025 | -1.446 | -0.523 |
| -> MBNMA | | -0.528 | -0.961 | -0.261 |
|  |  |  |  |  |
| Qigong_500 vs Placebo_0 | 0.402 |  |  |  |
| -> direct |  | -0.772 | -1.227 | -0.335 |
| -> indirect |  | -0.217 | -1.268 | 0.668 |
| -> MBNMA | | -0.689 | -1.088 | -0.271 |
|  |  |  |  |  |
| Qigong_250 vs Placebo_0 | 0.505 |  |  |  |
| -> direct |  | -0.269 | -1.337 | 0.819 |
| -> indirect |  | -0.65 | -1.104 | -0.21 |
| -> MBNMA | | -0.593 | -1.033 | -0.203 |
|  |  |  |  |  |
| Mix_1000 vs Placebo_0 | 0.476 |  |  |  |
| -> direct |  | -0.021 | -1.293 | 1.166 |
| -> indirect |  | -0.384 | -0.897 | 0 |
| -> MBNMA | | -0.332 | -0.792 | 0.011 |
|  |  |  |  |  |
| Mix_750 vs Placebo_0 | 0.671 |  |  |  |
| -> direct |  | -0.472 | -1.088 | 0.128 |
| -> indirect |  | -0.245 | -0.757 | 0.115 |
| -> MBNMA | | -0.317 | -0.703 | 0.011 |
|  |  |  |  |  |
| Mix_500 vs Placebo_0 | 0.641 |  |  |  |
| -> direct |  | -0.285 | -1.101 | 0.562 |
| -> indirect |  | -0.265 | -0.65 | 0.072 |
| -> MBNMA | | -0.297 | -0.649 | 0.011 |
|  |  |  |  |  |
| AE_1000 vs Placebo_0 | 0.025 |  |  |  |
| -> direct |  | -2.611 | -3.676 | -1.615 |
| -> indirect |  | -0.661 | -1.313 | -0.226 |
| -> MBNMA | | -1.118 | -1.763 | -0.453 |
|  |  |  |  |  |
| AE_750 vs Placebo_0 | 0.697 |  |  |  |
| -> direct |  | -0.842 | -1.708 | 0.066 |
| -> indirect |  | -1.093 | -1.612 | -0.456 |
| -> MBNMA | | -0.992 | -1.471 | -0.453 |
|  |  |  |  |  |
| AE_250 vs Placebo_0 | 0.231 |  |  |  |
| -> direct |  | -0.258 | -0.84 | 0.31 |
| -> indirect |  | -0.966 | -1.481 | -0.439 |
| -> MBNMA | | -0.574 | -1.028 | -0.304 |

**Figure 8.3.** Node-splitting analysis (density plot). The first value indicates the agent and the second one is the corresponding dose of that agent. *AE* Aerobic Exercise, *Mix* Mixed exercise, RT Resistance Training, *TC* Tai Chi.

Supplementary 9: Non-linear functions and models fit comparison

The different doses of exercise were meta-analysed as independent and unrelated treatments (i.e., “split” NMA). This step is useful to determine which function fits the data better and should subsequently be used in a Model-Based Network Meta-Analysis (MBNMA) (Pedder, 2021). Figure 9.1 show the different responses (SMD) of each dose for different treatments, respectively.

**Figure 9.1.** “Split” NMA of different exercise agents. *AE* Aerobic Exercise, *Mix* Mixed exercise, RT Resistance Training, *TC* Tai Chi.

Table 9.1 shows the fit indices from each of the models fitted. For our data, restricted cubic splines show the best fit and were therefore used in subsequent analyses.

**Table 9.1.** Models fit comparison

| **Model** | **DIC** | **SD** | **Deviance** | **Residual deviance** | **pD** |
| --- | --- | --- | --- | --- | --- |
| **Emax**  **(****common treatment effects)** | 483.5 | NA | 419.258 | 240.675 | 65.0 |
| **Emax**  **(RANDOM treatment effects)** | 403.6 | 0.528 | 299.933 | 121.350 | 104.3 |
| **Linear**  **(common treatment effects)** | 470.3 | NA | 407.966 | 229.383 | 63.0 |
| **Linear**  **(random treatment effects)** | 403.8 | 0.426 | 303.628 | 125.044 | 100.6 |
| **Exponential**  **(common treatment effects)** | 610.9 | NA | 548.370 | 369.787 | 63.3 |
| **Exponential**  **(RANDOM treatment effects)** | 404.9 | 0.603 | 298.310 | 119.726 | 107.0 |
| **Restricted cubic spline**  **(common treatment effects; 3 knots)** | 425.7 | NA | 350.672 | 172.088 | 75.2 |
| **Restricted cubic spline**  **(random treatment effects; 3 knots)** | 403.5 | 0.314 | 306.641 | 128.058 | 98.5 |
| **Non-parametric monotonically up**  **(common treatment effects)** | 1031.1 | NA | 974.809 | 796.225 | 56.9 |
| **Non-parametric monotonically up**  **(RANDOM treatment effects)** | 413.4 | 1.428 | 297.962 | 119.379 | 116.1 |

DIC = Deviance Information Criterion; SD = Between-study Standard Deviation; pD: Number of estimated parameters; NA = Not Applicable. The SD is presented as the main value and (95% Credible Intervals).

Further to model fit indices, deviance plots showing the contribution of each data point to the residual deviance are also useful to confirm the robustness of model selection (Pedder, 2021). Each data point should contribute about 1 to the posterior mean deviance, which indicates good model fit (Dias, Sutton, Ades, & Welton, 2013). The deviance plot for treatment effects (Supplementary Figure 9.1) confirm the robustness of our model selection.

**References**

Dias, S., Sutton, A. J., Ades, A. E., & Welton, N. J. (2013). Evidence synthesis for decision making 2: a generalized linear modeling framework for pairwise and network meta-analysis of randomized controlled trials. *Medical Decision Making : an International Journal of the Society For Medical Decision Making, 33*(5), 607-617. doi:10.1177/0272989X12458724

Pedder, H. (2021). *MBNMAdose: An R package for incorporating dose-response information into Network Meta-Analysis.* Paper presented at the Evidence Synthesis and Meta-Analysis in R Conference 2021.

Puhan, M. A., Schünemann, H. J., Murad, M. H., Li, T., Brignardello-Petersen, R., Singh, J. A., . . . Guyatt, G. H. (2014). A GRADE Working Group approach for rating the quality of treatment effect estimates from network meta-analysis. *BMJ (Clinical research ed.), 349*.

Ter Veer, E., van Oijen, M. G. H., & van Laarhoven, H. W. M. (2019). The Use of (Network) Meta-Analysis in Clinical Oncology. *Frontiers in Oncology, 9*, 822. doi:10.3389/fonc.2019.00822

van Valkenhoef, G., Dias, S., Ades, A. E., & Welton, N. J. (2016). Automated generation of node-splitting models for assessment of inconsistency in network meta-analysis. *Research Synthesis Methods, 7*(1), 80-93. doi:10.1002/jrsm.1167

Veroniki, A. A., Mavridis, D., Higgins, J. P. T., & Salanti, G. (2014). Characteristics of a loop of evidence that affect detection and estimation of inconsistency: a simulation study. *BMC medical research methodology, 14*, 106. doi:10.1186/1471-2288-14-106

Wheeler, D. C., Hickson, D. A., & Waller, L. A. (2010). Assessing Local Model Adequacy in Bayesian Hierarchical Models Using the Partitioned Deviance Information Criterion. *Computational Statistics & Data Analysis, 54*(6), 1657-1671. Retrieved from <https://pubmed.ncbi.nlm.nih.gov/21243121>
